# Shale-Gas-Related Induced Seismicity: Geospatial Patterns, Forecasting Approaches and Risk Governance

**DOI:** 10.3390/e28101059

**Published:** 2026-09-26

**Authors:** Jinhong Wang, Rui Wang, Peng Han, Weiwei Wu, Lingyuan Meng, Changsheng Jiang

**Affiliations:** 1 Department of Earth and Space Sciences, Southern University of Science and Technology, Shenzhen 518055, China; 2 Guangdong Provincial Key Laboratory of Geophysical High-Resolution Imaging Technology, Southern University of Science and Technology, Shenzhen 518055, China; 3 Shenzhen Key Laboratory of Deep Offshore Oil and Gas Exploration Technology, Southern University of Science and Technology, Shenzhen 518055, China; 4 Sichuan Earthquake Agency, Chengdu 610041, China; 5 China Earthquake Networks Center, Beijing 100045, China; 6 Institute of Geophysics, China Earthquake Administration, Beijing 100081, China

**Keywords:** induced seismicity, shale gas, hydraulic fracturing, wastewater disposal, maximum magnitude forecasting

## Abstract

As global energy systems pursue lower-carbon transition pathways, the rapid expansion of shale-gas development has increased subsurface fluid injection, intensifying concerns about induced seismicity and associated seismic risk. Although regional studies have documented specific induced-seismicity sequences, global patterns, regional variability, forecasting approaches, and governance practices have rarely been examined together within an integrated framework. This review synthesizes shale-gas-related induced seismicity across major production regions, including North America, Europe, and China, with particular emphasis on common patterns and contrasting seismic responses to hydraulic fracturing and wastewater disposal. Across diverse geological and operational settings, the reviewed evidence indicates that wastewater disposal is generally associated with larger-magnitude and more spatially widespread seismicity, whereas hydraulic fracturing typically produces localized seismicity but can also be associated with significant events where critically stressed faults are hydraulically connected to the stimulated zone. Building on this regional synthesis, we compare statistical, physics-based, hybrid, and machine-learning forecasting methods in terms of their operational applicability, predictive capabilities, data requirements, and limitations. However, translating these methodological advances into effective risk reduction requires their integration with robust monitoring and adaptive governance practices. Current governance measures can reduce risk, but their effectiveness depends on sufficiently sensitive monitoring, timely data exchange, regionally calibrated and adaptive operational thresholds, and continued post-injection surveillance. Future risk management should therefore move toward dynamic, physics-constrained geospatial frameworks that integrate seismic observations, injection histories, fault architecture, and regional stress conditions to support adaptive operational decision-making.

## 1. Introduction

Shale gas has become an important component of the global energy transition because of its large resource base, relatively lower carbon intensity than coal, and compatibility with existing natural-gas infrastructure. Its commercial development relies mainly on horizontal drilling and hydraulic fracturing, which enable the stimulation of low-permeability shale reservoirs and the economic recovery of tightly bound natural gas [1]. However, shale-gas development is accompanied by intensive subsurface fluid injection, wastewater handling, and reservoir disturbance. Hydraulic fracturing, wastewater disposal, and related industrial operations can alter pore pressure, poroelastic stress, and fault strength, thereby promoting the reactivation of pre-existing faults under favourable geological and tectonic conditions [2,3,4,5]. As a result, shale-gas-related induced seismicity has evolved from a local operational safety issue into a broader seismological problem involving fault instability, earthquake nucleation, public safety, and risk governance [2,3].

Shale-gas-related induced seismicity has been reported in several major production regions worldwide, but its characteristics vary substantially across tectonic and operational settings. In the central United States, long-term wastewater disposal has been associated with broad pore-pressure diffusion, basement-fault activation, and multiple moderate earthquakes in Oklahoma, Arkansas, and Texas [6,7,8,9,10]. In western Canada, many earthquake sequences are more directly linked to hydraulic fracturing, particularly where stimulation interacts with critically stressed faults, overpressured shale formations, or hydraulically connected fracture systems [11,12,13]. In the United Kingdom, the Preese Hall sequence demonstrated that even relatively small hydraulic-fracturing-induced earthquakes can have major regulatory consequences when they occur near previously unrecognised faults [14,15]. In the Sichuan Basin of China, moderate events and strong aftershock sequences have occurred in areas affected by shale-gas hydraulic fracturing, wastewater injection, salt mining, and regional tectonic loading, making causality assessment more complex [16,17,18,19,20]. These regional contrasts indicate that induced seismicity is not controlled by fluid injection alone, but by the coupled effects of operational forcing, fault architecture, basement proximity, reservoir permeability, regional stress state, and monitoring capability.

Previous studies have addressed shale-gas-induced seismicity from mechanistic, forecasting, and risk-management perspectives. Proposed triggering mechanisms include pore-pressure diffusion, poroelastic stress transfer, static Coulomb stress changes, aseismic slip, and the reactivation of pre-existing faults [2,3,4,5]. Statistical and volume-based approaches have been used to estimate seismicity rates and maximum magnitudes, whereas physics-based models attempt to represent pressure diffusion, rate-and-state earthquake nucleation, and stress evolution on faults [21,22,23,24,25]. Hybrid physical-statistical models have further improved the capacity to forecast time-dependent and spatially variable induced-seismic hazard under changing injection scenarios [21,25]. Risk-management measures, including dense seismic monitoring, injection adjustment, operational suspension, traffic-light systems, and post-event regulatory review, have also been implemented in several regions [3,15,26]. Nevertheless, current knowledge remains fragmented. Many studies focus on individual earthquake sequences, specific basins, or single operational mechanisms, whereas systematic comparisons across shale-gas regions remain limited. In particular, hydraulic-fracturing-induced and wastewater-disposal-induced seismicity are often discussed together, despite substantial differences in injection duration, cumulative injection volume, spatial footprint, pressure evolution, and maximum-magnitude potential [8,12,13,27].

This review includes studies published primarily between 2000 and 2024 and identified through Web of Science, Scopus, and Google Scholar using combinations of terms including “shale gas”, “hydraulic fracturing”, “wastewater disposal”, “induced seismicity”, “injection-induced earthquakes”, “maximum magnitude”, “seismicity-rate forecasting”, “geospatial susceptibility”, “traffic-light system”, “seismic monitoring”, and “risk governance”. Earlier seminal studies were also included when they introduced fundamental concepts, documented important historical cases, or established widely used methodological frameworks. Event and operational information was supplemented using the Human-Induced Earthquake Database (HiQuake), USGS resources, and reports from governmental and regulatory agencies. Studies were screened for their relevance to geological and operational controls, triggering mechanisms, forecasting methods, monitoring, or risk governance. Representative cases were selected to cover major shale-gas-producing regions, different operation types, geological settings, injection characteristics, maximum observed magnitudes, and proposed triggering mechanisms. This procedure was intended to provide a transparent and representative synthesis rather than an exhaustive systematic review.

Based on this literature and case-selection framework, this provides a global synthesis of shale-gas-related induced seismicity, with emphasis on spatial distribution patterns, regional variability, triggering mechanisms, forecasting approaches, and risk-management practices. By examining representative cases from North America, Europe, and China, this review compares seismic responses associated with hydraulic fracturing and wastewater disposal, and evaluates the applicability of statistical, physics-based, hybrid, and data-driven approaches to seismicity-rate forecasting and maximum-magnitude estimation. Finally, we discuss how dense seismic monitoring, data sharing, traffic-light systems, and geospatial susceptibility mapping can support dynamic and spatially explicit risk governance in shale-gas development regions [3,15,26,27].

## 2. Shale-Gas-Related Induced Seismicity: Spatiotemporal Patterns and Operational Types

Induced seismicity associated with shale gas development exhibits pronounced spatial heterogeneity worldwide. Figure 1 shows the major shale gas development regions within the global distribution of reported anthropogenic seismicity. North America has one of the highest concentrations of reported cases. In Oklahoma and other parts of the central United States, reported earthquake sequences are primarily associated with wastewater disposal [7,8,9,10]. In western Canada, particularly Alberta and British Columbia, many sequences are associated with hydraulic fracturing [11,12,13]. The Sichuan Basin in China is another major concentration of shale gas-related induced seismicity and a key focus of research [18]. Relatively few cases have been reported in Europe. Hydraulic fracturing-related earthquakes have been documented in the United Kingdom, whereas the association between very shallow earthquakes and hydraulic fracturing operations near Wysin, Poland, remains uncertain [14,28]. Regional differences in seismic network detection capabilities, catalog magnitude completeness and temporal coverage, and event attribution and reporting practices may influence case identification and database inclusion.

Concerns about industrially induced seismicity predate large-scale shale gas development. In the 1960s, deep-well waste fluid injection at the Rocky Mountain Arsenal near Denver was followed by a well-documented earthquake sequence [30]. Although unrelated to shale gas, this operation provided an important early case for studying the seismic effects of fluid injection. Early observations focused largely on mining-related earthquakes and surface deformation in oilfields [23,31,32]. Research later expanded to seismicity associated with reservoir impoundment, deep fluid injection and conventional natural gas production [30,33,34,35]. The combination of horizontal drilling and hydraulic fracturing subsequently enabled large-scale unconventional resource development, expanding reservoir stimulation and wastewater management activities in producing regions [2,8,9,10,11]. Rising seismicity in Oklahoma and hydraulic fracturing-related earthquake sequences in Alberta and Sichuan brought wider scientific and regulatory attention to the issue [8,18,36].

Figure 2 places these developments in a longer historical context. Records of industrially induced seismicity were sparse before the mid-twentieth century. Reported cases increased from the 1960s onwards, broadly coinciding with sustained attention to deep-well injection, reservoir impoundment and conventional oil and gas development. Case counts rose further after 2000, with a marked increase from the late 2000s to the mid-2010s. Shale gas-related cases were particularly concentrated in this period, indicating that associated seismic concerns emerged rapidly as unconventional oil and gas development expanded. Annual reported case counts subsequently declined from their peak but remained well above earlier levels. Annual maximum magnitudes, meanwhile, fluctuated considerably throughout the record and did not track changes in reported case counts. The oil price series in Figure 2 provides economic context only; coincident changes over time do not establish a causal effect on seismicity.

The spatial clustering and temporal trends described above may reflect the combined effects of hydraulic fracturing and wastewater disposal during shale gas development, rather than a single operational process. Hydraulic fracturing (HF) and wastewater disposal (WD) differ in their operational objectives, injection pressures, duration, cumulative injection volumes and target formations (Table 1). Distinguishing between these operations helps interpret the observed spatiotemporal patterns and their possible causes.

Hydraulic fracturing uses staged injection of fluid and proppant along horizontal wells to create or extend fracture networks, improving flow through low-permeability shale reservoirs [37,38,39,40,41]. Injection is typically brief and rapid, with pressures that may exceed the formation breakdown pressure. Seismic responses are commonly analyzed with respect to stage locations and treatment schedules. Most monitored operations produce predominantly microearthquakes, but interactions with favorably oriented pre-existing faults close to failure can trigger larger, felt earthquakes [2,42,43,44]. Wastewater disposal injects saline waste fluids, including flowback and produced water, into subsurface formations and differs from injection used to maintain production reservoir pressure [35,37,45,46]. Disposal typically continues for months or years, potentially generating more extensive and persistent pore-pressure perturbations. Associated seismicity may extend beyond the vicinity of disposal wells and continue after injection ceases. Its extent and duration depend on hydraulic connectivity, formation permeability, injection history and fault conditions [8,10,14]. Both activities can promote fault slip by increasing pore pressure and reducing effective normal stress, or by altering stresses in the surrounding rock [2,5]. Fault responses also depend on orientation, initial stress, hydraulic connectivity and the evolution of pressure and stress perturbations [12,13,47]. A single operational parameter, therefore, cannot fully explain differences in seismic response.

**Table 1:** Industrial activities related to shale-gas-induced seismicity.

	Hydraulic Fracturing (HF)	Wastewater Disposal (WD)
Purpose	Artificially create fracture networks within hydrocarbon-rich source rocks to improve shale permeability and enhance gas recovery.	Safely dispose of high-salinity wastewater generated during shale gas production by long-term underground injection, thereby reducing surface treatment pressure and minimizing environmental pollution risks.
Injected fluid composition	Fracturing fluid consisting of water (~94%), inert solid materials (proppants, ~5%), and chemical additives (~1%).	Produced water and/or flowback fluid, often characterized by high salinity, containing formation-derived constituents and potentially residual hydraulic-fracturing additives and chemicals used during production operations, such as corrosion inhibitors, scale inhibitors, and biocides [48,49].
Injection method and pressure	Short-term, high-pressure, rapid injection (exceeding the formation breakdown pressure).	Long-term, medium-pressure, stable injection (below the formation breakdown pressure).
Target formation characteristics	Low-permeability shale formations with high organic matter content.	Reservoir formations with relatively high permeability that can readily accommodate and transmit injected fluids, such as limestone or sandstone.

Early microseismic observations indicate that seismic responses to hydraulic fracturing (HF) are generally dominated by small events. Warpinski [42] reviewed monitoring data from thousands of fracturing treatments, in which most recorded magnitudes were well below M1.0. During 2009–2011, Natural Resources Canada reported 38 earthquakes in the Horn River investigation area, only one of which was reported as felt. The investigation also noted that more than 8000 hydraulic fracturing completions in northeastern British Columbia had no associated anomalous seismicity identified [50]. These findings describe the dominant responses in monitored operations but do not establish an upper magnitude limit for HF-related earthquakes under other geological conditions. Larger events remain possible where fracturing perturbations reach favorably oriented pre-existing faults that are close to failure and hydraulically connected to the injection interval. Subsequent observations in western Canada, Sichuan and the United Kingdom show that HF-related seismicity extends beyond microearthquakes, with recorded events exceeding M3.0 and some exceeding M4.0 [18,44]. For example, an $M_{w}4.6$ earthquake occurred near Fort St. John, British Columbia, on 17 August 2015 [51]. These cases prompted closer examination of how hydraulic connectivity and stress transfer transmit stimulation-induced perturbations to faults capable of larger ruptures [12,13,52]. The Preston New Road case in the United Kingdom also shows that seismicity can continue after pumping stops [53]. HF-related seismic hazard should therefore be assessed in relation to operational history, fault conditions and connections between faults and the injection interval. The predominance of small events does not exclude larger earthquakes. Nor should assessments assume a universal relationship between magnitude and injection depth, or that seismic hazard disappears immediately after pumping stops.

Studies of wastewater disposal indicate that sustained injection can increase regional seismicity. Walsh and Zoback [10] found that disposal rates increased five- to tenfold across three study areas in Oklahoma, accompanied by substantial increases in seismicity. However, temporal coincidence alone cannot establish a causal link between individual earthquakes and specific disposal wells. Moreover, the wastewater originated mainly from conventional oil production, so these earthquakes cannot all be attributed to shale gas development. Modelling by Keranen et al. [8] suggested that pressure perturbations from high-rate injection wells could propagate approximately 35 km and potentially trigger faults close to failure. However, the propagation distance and triggering pressure depend on model assumptions and geological conditions. The Prague, Fairview and Pawnee sequences further demonstrate that wastewater disposal-related earthquakes can exceed M5.0, with the Pawnee earthquake reaching $M_{w}5.8$ [54,55,56,57]. Seismic hazard assessments should therefore consider cumulative injection history, pressure propagation and fault connectivity. Attribution should not rely solely on the distance between an earthquake and the nearest disposal well. To compare the seismic responses associated with these two activities, Table 2 brings together representative cases, classified by operation type and certainty of attribution.

## 3. Regional Characteristics of Shale Gas–Induced Seismicity

Shale gas development, accompanied by hydraulic fracturing and wastewater injection, has induced significant seismic activity in regions such as North America and Asia. Although the underlying physical mechanisms of these induced earthquakes are broadly universal, their manifestations, societal impacts, and regulatory responses vary considerably across regions due to differences in geological conditions, energy development models, and policy frameworks. The following section, therefore, adopts a regional perspective to systematically review the characteristics of induced seismicity in major shale gas production areas of North America, Europe, and Asia.

### 3.1. United States

As the pioneer of global shale gas development, North America has concentrated its production primarily in the United States and Canada. Leveraging advanced hydraulic fracturing and horizontal drilling technologies, both countries have accelerated the commercialization of unconventional oil and gas resources, thereby driving the transition of the energy structure toward a natural gas–dominated system. However, with the continued intensification of development, induced seismicity has emerged as a growing concern, attracting significant attention from both the public and the academic community. Institutions such as the United States Geological Survey and Natural Resources Canada have established high-density seismic monitoring networks, which have advanced research in mechanism identification, magnitude prediction, and hazard assessment of induced earthquakes. These efforts have also facilitated the implementation of various risk-mitigation measures, including magnitude-threshold controls and injection-rate restrictions.

The distribution of shale gas resources in the United States is characterized by an “abundant in the east and sparse in the west” pattern (Figure 3), with production concentrated primarily in several major basins and scattered occurrences elsewhere. The principal production areas include the Marcellus and Utica shales in the Appalachian Basin, the Barnett shale in the Fort Worth Basin, the Haynesville/Bossier shales in the western Gulf of Mexico Basin and the Texas–Louisiana Salt Basin, the Woodford shale in the Arkoma and Anadarko Basins, and the Fayetteville shale in the Arkoma Basin [78,79]. According to projections by the United States Energy Information Administration (EIA), shale gas will account for 46% of the nation’s natural gas supply by 2035 [80]. However, with the continued expansion of extraction, seismic frequency and magnitudes have increased markedly in regions such as Oklahoma, Texas, Colorado, and New Mexico. This phenomenon is widely attributed to high-pressure fluid injection and wastewater disposal practices [22,44,81].

The United States drilled its first natural gas well as early as 1821, but the true commercialization of shale gas development began in the late 1990s, marked by the technological breakthroughs of Mitchell Energy in the Barnett play of the Fort Worth Basin. This milestone enabled the first economically viable hydraulic fracturing operations and successfully launched the so-called “shale gas revolution,” which has since become a paradigm for global unconventional oil and gas exploration and development. In the 21st century, with the rapid expansion of the shale gas industry, seismic activity in the United States has shown a marked acceleration, particularly after 2009. According to data from the United States Geological Survey (USGS) Preliminary Determination of Epicenters and the National Seismic Hazard Map, the average annual rate of earthquakes of M≥3 was 21±7.6 events between 1970 and 2000, rising to 29±3.5 events during 2001~2008. After 2009, however, the number of M≥3 events increased dramatically: 50 in 2009, 87 in 2010, and 134 in 2011. Taking Oklahoma as an example, the long-term annual average of M≥3 earthquakes prior to 2009 was only 1.2, but since 2009 it has surged to more than 25 per year. USGS seismologists have noted that such a sharp increase in seismicity is virtually unprecedented in non-volcanic regions or in the absence of a major triggering mainshock, concluding that this growth is “almost certainly” attributable to anthropogenic activities [2].

#### 3.1.1. The Appalachian Basin (The Utica and Marcellus Shales)

In the Northern Appalachian Basin of the eastern United States, the organic-rich Ordovician Point Pleasant–Utica and the Middle Devonian Marcellus shales constitute one of the most developed unconventional hydrocarbon plays in the world. This region has become both the largest and fastest-growing shale gas–producing area in the United States [82]. Accompanying this rapid development, seismic activity associated with hydraulic fracturing and wastewater injection has gradually increased, making induced earthquakes an important geological phenomenon accompanying unconventional resource exploitation in this region [58,83].

The Appalachian Basin has experienced relatively fewer and more isolated seismic events, which are mainly concentrated along a north–south-trending seismic belt across eastern Ohio, western Pennsylvania, and central West Virginia, and this spatial variability may be linked to differences in local geological conditions and shale gas development targets [68]. Specifically, Ohio has recorded a significantly higher frequency of seismicity than neighboring Pennsylvania and West Virginia. This difference is partly attributed to the fact that many disposal wells in Ohio are located closer to the Precambrian basement and that the state has a relatively larger number of disposal wells, and partly to the deeper burial of the Utica–Point Pleasant shale formations being exploited, which lie nearer to the geological basement and therefore more easily activate basement fault systems [84,85].

The first clearly documented case of hydraulic fracturing–induced seismicity in Ohio occurred in Harrison County, followed by multiple similar events in adjacent areas [83,86]. Statistics show that in regions where hydraulic fracturing–induced earthquakes occur frequently, approximately 10% to 33% of fracturing wells are capable of generating detectable seismicity. Seismological analyses, including double-difference relocation and focal mechanism inversion, revealed that these events are mainly concentrated on a series of linearly distributed strike-slip fault segments. Although these faults do not correspond to those mapped on existing geological surveys, their spatial consistency suggests that they represent latent structures susceptible to failure under the regional stress field. The stronger induced earthquakes typically occur near the Precambrian basement, with the largest documented event ($M_{L}3.7$) occurring on 3 June 2017, which has been conclusively linked to hydraulic fracturing operations in the Utica Shale [83].

#### 3.1.2. The Anadarko and Arkoma Basins (The SCOOP/STACK Plays and Fayetteville Formation)

The Anadarko and Arkoma Basins, located in the south-central United States, have emerged as important centres of unconventional oil and gas development in recent years. Within these basins, the SCOOP (South Central Oklahoma Oil Province) and STACK (Sooner Trend Anadarko Canadian Kingfisher) plays have become core areas of the shale oil and gas industry due to their superior reservoir quality and significant development potential. These plays encompass multiple prospective formations, most notably the Late Devonian Woodford Shale and Mississippian carbonates, which are characterized by low permeability. Both SCOOP and STACK are situated in Oklahoma, spanning the western portions of the Anadarko and Arkoma Basins. The region is structurally complex, and with the intensive application of hydraulic fracturing and horizontal drilling technologies, seismic activity has increased markedly. Consequently, this area has become a representative case for investigating the mechanisms of induced seismicity associated with hydraulic fracturing and wastewater injection.

Before 2009, Oklahoma experienced only 1–2 earthquakes of magnitude 3 or greater per year. Such a low level of seismicity reflected the fact that Oklahoma is neither a tectonically active region nor located near a plate boundary. However, by 2015 this number had surged to more than 900 events. Between 2008 and 2013 alone, Oklahoma accounted for 45% of all M≥3.0 earthquakes in the central and eastern United States. This dramatic increase in seismicity is commonly attributed to the deep injection of wastewater generated during shale gas development. Although the overall seismicity rate has declined since 2016, the cumulative seismic moment release has remained high, largely due to the occurrence of multiple strong earthquakes (M≥5.0) between 2011 and 2016. These events include the Prague earthquake on 5 November 2011 ($M_{w}5.7$) [54,55], the Fairview earthquake on 13 February 2016 ($M_{w}5.1$) [56,87], the Pawnee earthquake on 3 September 2016 ($M_{w}5.8$) [88,89], and the Cushing earthquake on 7 November 2016 ($M_{w}5.0$) [57,90]. The sharp escalation in seismic activity has raised widespread public concerns regarding the safety of local residents and the stability of critical infrastructure.

In comparison with other areas of the central and eastern United States, Oklahoma has experienced a disproportionately high rate of seismicity, a phenomenon attributed mainly to wastewater injection, with hydraulic fracturing contributing to a lesser extent [10,91]. In recent years, studies have shown that both the spatial extent and scale of induced seismicity in Oklahoma have expanded significantly. Using an enhanced earthquake catalogue generated by multi-station template matching between 2010 and 2016, together with all available hydraulic fracturing information, a total of 274 wells were identified as being associated with seismic activity, with the largest event being an $M_{L}3.5$ earthquake on 14 July 2015 [92]. Within the SCOOP and STACK plays alone, approximately 130 hydraulic fracturing wells were spatially and temporally correlated with more than 700 events of $M_{L}\geq 2.0$ between 2010 and 2016. It was found that more than 75% of seismic activity could be directly attributed to local hydraulic fracturing wells, with this proportion exceeding 95% in some areas, and over half of all fracturing wells were capable of inducing detectable seismic events. Research on wastewater injection–induced earthquakes in central Oklahoma further indicates that most events occur on near-vertical strike-slip faults in the shallow crystalline basement at depths of less than 6 km. These reactivated fault segments are primarily oriented northeast or northwest, consistent with the regional compressive stress field trending approximately N85° E [90].

Located in the eastern segment of the Arkoma Basin, the Fayetteville Shale is a typical tight shale gas reservoir and represents the second shale gas play in the United States to achieve commercial development. It is bounded by the Ozark Plateau to the north, the Ouachita Fold Belt to the south, the Southern Oklahoma Basin to the west, and the Reelfoot Rift to the east [93]. Production began in 2004 with 14 vertical wells, and by 2012, Fayetteville shale gas output had reached its peak. During the same period, seismic activity in the region increased, prompting Arkansas to initiate preliminary regulatory measures. To date, more than 2000 hydraulic fracturing wells have been completed in the state.

Seismicity in Arkansas is generally characterized by low magnitudes, with the most significant historical events concentrated in the New Madrid Seismic Zone in the northeastern part of the state. Earthquake swarms occurred near Enola, Arkansas, in 1982 and 2001, before wastewater disposal began in the vicinity. These earlier sequences should be distinguished from the 2010–2011 Guy–Greenbrier earthquake sequence associated with wastewater injection [94,95,96,97,98]. Beginning on 23 September 2010, intense earthquake swarm activity was detected near the Guy-Greenbrier fault. Comparisons between modeled spatiotemporal pore-pressure variations and observed seismic activity indicated that minimal pore pressure increases of approximately 0.009 and 0.035 MPa were sufficient to trigger seismicity in the basement and sedimentary portions of the fault, respectively [96]. The Guy–Greenbrier sequence primarily involved slip on faults within the crystalline basement [7]. In February 2011, seismic activity migrated further south, extending the seismically active segment of the Guy-Greenbrier fault to approximately 13 km. Following the largest earthquake of the sequence, an $M_{w}4.7$ event on 28 February 2011, the Arkansas Oil and Gas Commission (AOGC) issued an emergency order suspending wastewater injection operations due to concerns over the potential for larger earthquakes. Systematic analyses of the swarm activity, injection patterns, and pore pressure diffusion processes indicate that wastewater disposal was most likely responsible for reactivation of the Guy-Greenbrier fault [96,97,98]. Similarly, in the neighboring states of Oklahoma and Texas, wastewater injection has been widely confirmed as a major driver of induced regional seismicity [2,54,99].

#### 3.1.3. The Western Gulf Basin (The Eagle Ford Shale Play)

The Western Gulf Basin, located in southern Texas, is an important component of the Gulf of Mexico coastal sedimentary system. Within this basin, the Eagle Ford Shale is one of the most significant shale oil and gas production zones in North America, characterized by favourable attributes such as moderate burial depth, high organic matter content, and appropriate thermal maturity, making it highly suitable for horizontal drilling and hydraulic fracturing. The Eagle Ford Shale is primarily distributed across southern Texas, extending from the Mexican border to the southeastern coastal area, covering an area of more than 100,000 km^2^ [100].

With the large-scale application of hydraulic fracturing and well completion technologies, a number of earthquakes related to fluid injection have been recorded in parts of the Eagle Ford play. Monitoring data indicate that seismic activity corresponds closely in time and space with operational schedules, displaying typical characteristics of induced seismicity. The triggering mechanisms are mainly attributed to elevated pore pressure, stress perturbations, and reactivation of critically stressed faults caused by fracturing, while a small number of stronger earthquakes are thought to be associated with deep wastewater disposal [101]. Elsewhere in Texas, the $M_{w}4.8$ earthquake near Timpson on 17 May 2012 was investigated as an event possibly triggered by wastewater injection [81]. By 2018, the rate of earthquakes of $M_{L}\geq 3.0$ in the Eagle Ford region had reached 33 times the historical background level (only three events per decade between 1980 and 2010). Systematic analyses of seismicity since 2014, using template matching techniques, have further examined the spatiotemporal associations between hydraulic fracturing and earthquake occurrence. Results show that more than 85% of seismic events in the region coincided closely with hydraulic fracturing activities, with 94 earthquakes of $M_{L}\geq 2.0$ being clearly correlated with 211 fracturing stages [102]. By contrast, wastewater injection operations in this area have not exhibited significant spatiotemporal correlations with seismicity.

#### 3.1.4. The Permian Basin (The Wolfcamp and Bone Spring Formations Permian)

The Permian Basin, located in the southwestern United States and spanning western Texas and southeastern New Mexico, is one of the most prolific hydrocarbon provinces in the country. Within it, the Delaware Basin in the western part represents one of the most important sub-basins, characterized by great sedimentary thickness, high pressure gradients, and abundant organic matter. The primary reservoirs are concentrated in the Wolfcamp and Bone Spring formations. The Bone Spring Formation, situated beneath the Delaware Mountain Group, consists of calcareous, siliceous, and carbonaceous sedimentary rocks rich in organic matter [103]. Underlying this, the Wolfcamp Shale is widely regarded as a highly prospective, prolific tight oil and gas reservoir.

Oil and gas development has been ongoing in this region since the 1930s, and most earthquakes recorded here are believed to have been induced by industrial activities [104,105,106]. In recent years, seismicity in the Delaware Basin has increased markedly, particularly in the southern portion of the basin [107,108]. No earthquakes exceeding magnitude 3.0 were recorded in the southern Delaware Basin before 2015. Between 2015 and 2020, however, more than 67 such events occurred, including the $\;M_{w}5.0$ Mentone earthquake of 26 March 2020. Most of the seismicity has been attributed to wastewater disposal, whereas approximately 5% of the earthquakes in the catalogue, corresponding to about 1850 events, were spatially and temporally associated with active hydraulic-fracturing operations [108,109]. Nevertheless, some studies suggest that hydraulic fracturing may represent one of the primary driving mechanisms behind these earthquakes. The southwestern Delaware Basin has emerged as the most representative area of hydraulic fracturing–induced seismicity, where the limited scale of wastewater disposal highlights the stronger correlation between fracturing operations and earthquake occurrence [108,110]. Given the relatively high frequency of seismic activity in this region, the coexistence of multiple potential drivers—including wastewater injection and oil and gas extraction—and the lack of publicly available detailed data on hydraulic fracturing operations, the actual scale of hydraulic fracturing–induced seismicity in the Delaware Basin is likely underestimated.

### 3.2. Canada

Canada is the only country outside the United States in North America to have achieved commercial shale gas production. With the intensification of shale gas development, seismic activity in Canada has shown an increasing trend, most notably in the Horn River Basin of northeastern British Columbia and the Fox Creek area of Alberta, located at the boundary with the Western Canada Sedimentary Basin (WCSB). Unlike in the central United States, where induced earthquakes are primarily associated with wastewater injection, induced seismicity in western Canada is predominantly linked to hydraulic fracturing operations, particularly to localized stress perturbations generated during high-pressure fluid injection in the well completion stage, which act as a key mechanism for reactivating faults and triggering earthquakes [111]. The Horn River Shale, the Montney trend, and the Fox Creek area together represent the three most intensive shale gas development zones and the most seismically active hotspots in Canada, as well as the core regions where research and regulatory policies on induced seismicity have been most extensively concentrated.

#### 3.2.1. The Horn River Basin (Horn River Formation/the Muskwa Formation/Upper Besa River Formation)

The Horn River Basin (HRB), located in northeastern British Columbia, is the earliest region in Canada to achieve large-scale commercial shale gas development. Within this basin, the Horn River Group shales, together with associated mudstones, the Muskwa Formation, and the upper part of the Besa River Formation, exhibit total organic carbon (TOC) contents of up to 5.7 wt.%, making them prime targets for shale gas exploration [112].

Preliminary signs of induced seismicity in the region were documented as early as the 1970s [113], and subsequent hydrocarbon development further increased both the frequency and magnitude of earthquakes. In the 1980s, the Fort St. John area recorded an event as large as $M_{L}4.3$, which was attributed to fluid injection operations [113]. Thereafter, with the decline in oil and gas production, regional seismicity subsided until shale gas redevelopment once again reactivated the local fault systems. Based on operational data compiled by the BCOGC, a hydraulic fracturing parameter database was established and cross-referenced with the regional seismic catalogue to quantitatively assess the relationship between fracturing activities and seismicity. The results indicated that elevated local seismicity levels were positively correlated with injection volume, but showed no significant relationship with injection pressure [114]. Further research revealed that in areas of intensive hydraulic fracturing, the number of earthquakes increased from 24 events prior to hydraulic fracturing (2002–2003) to 131 events in 2011, with the maximum magnitude rising from $M_{L}2.9$ to $M_{L}3.6$. The overall *b*-value was estimated at 1.21, higher than the average value of ~1.0 for tectonic/natural earthquakes, reinforcing the likelihood of a physical link between hydraulic fracturing operations and induced seismicity in the Horn River Basin [66].

#### 3.2.2. The Alberta Basin (The Duvernay Formation)

The Alberta Basin, located in western Canada, is one of the most important sedimentary basins in North America and has long been renowned for its abundant conventional and unconventional hydrocarbon resources. The Duvernay Formation, a widespread, highly mature, and organic-rich shale unit within the basin, has become one of the most systematically and extensively studied sites worldwide for hydraulic fracturing–induced seismicity. By 2016, more than 290 horizontal wells had been completed in the Duvernay Formation at depths ranging from 2.6 to 4.0 km, marking the onset of an intensive phase of unconventional development and drawing increased attention to the evolution of seismicity in the region.

Since the 1970s, multiple cases of induced seismicity associated with oil and gas extraction have been documented in central Alberta [115,116]. With the expansion of wastewater disposal operations, regional seismicity intensified, becoming particularly prominent after 2013. During this period, a sequence of earthquakes known as the “Crooked Lake Sequence” (CLS) emerged, with epicenters initially concentrated near Crooked Lake, approximately 30 km west of Fox Creek. These events were closely linked to hydraulic fracturing operations [117,118,119]. The CLS exhibited marked persistence and activity. Between 1 December 2013, and 31 December 2014, the Crooked Lake area recorded a series of earthquakes ranging from $M_{w}1.7$ to 3.9, with the largest being an $M_{w}3.9$ event on 23 January 2014. Subsequently, both the frequency and magnitude of induced earthquakes increased further: on 9 August 2014, an $M_{w}3.8$ earthquake occurred near Rocky Mountain House; on 23 January 2015, an $M_{L}4.4$ event was recorded near Crooked Lake; on 12 January 2016, an $M_{L}4.8$ earthquake struck the Fox Creek area; and on 4 March 2019, an $M_{L}4.18$ earthquake occurred near Red Deer in the Duvernay East Shale Basin and was associated with hydraulic fracturing [69]. Given the potential risks posed by the continued occurrence of moderate-magnitude earthquakes to regional infrastructure and public safety, the Alberta Energy Regulator (AER) revised and strengthened its Traffic Light Protocol (TLP) in 2019 to enhance real-time monitoring and risk control of hydraulic fracturing–induced seismicity.

Using data from regional seismic networks and local dense arrays, the characteristic features of induced earthquakes in the Duvernay play have been systematically revealed. These events are predominantly characterized by strike-slip focal mechanisms and systematically shallower focal depths compared with natural seismicity in the region [12,120,121,122,123]. Moreover, their occurrence frequency across a broad area has exceeded that of tectonic earthquakes [11], further confirming the strong spatiotemporal correlations between hydraulic fracturing operations and the Crooked Lake Sequence (CLS) swarm. In the Fox Creek area, Bao and Eaton [12] analyzed the relationship between fluid injection and seismic responses at six fracturing pads, finding that the largest event ($M_{w}3.9$) was associated with a fault potentially extending into the crystalline basement. The continued evolution of seismicity around this fault suggests that it may have been activated by industrial fluid injection. However, the specific slip and rupture mechanisms of this fault have not yet been clearly verified through forward modelling. Building on this, Gao et al. [124] proposed a more complex fluid diffusion and triggering mechanism: injected fluids initially spread eastward along interlayer pathways within the Duvernay Formation, inducing aseismic slip upon reaching the eastern fault, which subsequently led to deeper seismic rupture. High-precision hypocenter relocation further suggested that this pathway connected the eastern and western bounding faults near the crystalline basement, with its western end corresponding to the rupture point of the $M_{w}3.9$ event. This model provides new theoretical perspectives and supporting evidence for understanding far-field fault activation induced by hydraulic fracturing. Similarly, Eyre et al. [125] based on multi-source data from an $M_{w}4.1$ earthquake in Alberta, emphasized the central role of aseismic slip in induced earthquake processes.

From a geological perspective, induced earthquakes in the Duvernay play preferentially occur along the edges of carbonate reef platforms near the basement, which are considered sensitive zones for fluid migration and fault connectivity [126,127,128]. To further clarify the geological controls influencing induced seismic susceptibility in this region, researchers have applied machine learning methods to evaluate seismic sensitivity systematically. Several key variables were identified, including the distance between the injection horizon and the basement, regional stress conditions, overpressure levels, proximity to fossil reefs, lithium concentrations in groundwater, and the background seismicity rate. These factors provide a scientific basis for spatial prediction and susceptibility assessment of induced seismicity [129,130].

#### 3.2.3. The Western Canada Sedimentary Basin (The Montney Formation)

The Western Canada Sedimentary Basin (WCSB) is one of the most important hydrocarbon provinces in North America, spanning large areas of British Columbia and Alberta. It is characterized by thick sedimentary successions, complex tectonic evolution, and diverse petroleum systems. The Montney Formation, deposited during the Triassic period, comprises interbedded tight sandstones and shales with favorable reservoir properties and organic-rich source rocks, making it a key target for unconventional oil and gas exploration and development in recent years. Its development followed shortly after that of the Horn River Basin, but has been accompanied by an increase in the frequency and intensity of seismic activity. Between 2013 and 2014, a total of 231 earthquakes were recorded in the Montney region, with magnitudes ranging from $M_{L}2.2$ to 2.4. Of these, 193 events were closely correlated in time and space with hydraulic fracturing activities, while only 38 were attributed to wastewater disposal [43]. This contrast further underscores the hydraulic fracturing–dominated mechanism of induced seismicity in Canada.

Within the Montney play of British Columbia, the Montney North and Kiskatinaw areas represent two major seismic hotspots, displaying distinct spatial distributions and response characteristics of induced seismicity [43,131,132]. Focal mechanism analyses indicate that earthquakes in both regions are predominantly of thrust and strike-slip types. In Montney North, seismic events typically occur with a delay of about two days after hydraulic fracturing operations, with *b*-values ranging between 1.0 and 1.3. By contrast, in the Kiskatinaw area, located south of Fort St. John, the *b*-values are relatively higher (~1.5), indicating a larger proportion of small-magnitude events, most of which occur during or shortly after injection operations [131,133]. The largest induced earthquake recorded in this region to date was the $M_{w}4.6$ event that occurred in Montney North on 17 August 2015 [131,134].

### 3.3. Europe

Assessments by the EIA identify substantial technically recoverable shale gas resources in several European countries, including Poland, France, and Ukraine, although resource estimates remain uncertain and should not be equated with commercially recoverable reserves [135,136]. Potential shale-gas targets include Lower Paleozoic successions in the Baltic Basin, particularly Ordovician–Silurian shales in Poland, and Carboniferous Bowland–Hodder shales in England [137]. Other formations assessed for unconventional hydrocarbon potential include the Lower Jurassic Posidonia Shale and Oligocene Fish Shale in the Upper Rhine Graben, as well as the Upper Jurassic Mikulov Marl in the Vienna Basin [137]. European assessments have documented exploration activity in several countries while highlighting geological uncertainty, relatively high development costs, population density, environmental requirements, and public acceptance as constraints on commercial development [136,137,138].

Although large-scale commercial shale gas development has not been achieved in Europe, hydraulic fracturing in certain localities has clearly demonstrated the potential for induced seismicity, with the most representative case being a shallow microseismic sequence associated with fracturing operations near Blackpool, UK [139]. In 2011, Cuadrilla Resources conducted hydraulic fracturing at the Preese Hall well near Blackpool, during which 52 earthquakes were recorded and the maximum magnitude reached $M_{L}$2.3. Clarke et al. [14] attributed the sequence to the interaction of hydraulic fractures with a previously unidentified fault that was favorably oriented for reactivation under the local stress field. Following the suspension of shale-gas hydraulic fracturing after the 2011 Preese Hall events, the UK government permitted its resumption in December 2012, subject to additional controls on induced seismicity [140]. Hydraulic fracturing subsequently resumed at Preston New Road in 2018. The PNR-1z well targeted the Bowland Shale at a depth of approximately 2300 m, with an approximately east–west horizontal section extending about 700 m. Stimulation was conducted between October and December 2018 [68]. Seismicity was recorded by a surface monitoring network and a downhole geophone array installed in the adjacent PNR-2 well [141] The largest event during the 2018 campaign was reported as $M_{L}$1.6 in subsequent studies [53,142]. During this campaign, seismicity rates generally increased during injection and declined afterward, although the response varied substantially among stimulation stages. Stages near the eastern end of the horizontal section produced more seismicity than those near the western end despite comparable injection volumes [68]. Microseismic observations revealed interactions between stimulation and pre-existing structures [141], while modeling indicated that elastic stress transfer from opening hydraulic fractures contributed to the spatial distribution of PNR-1z seismicity [143].

Hydraulic fracturing of the adjacent PNR-2 well was conducted from 15 to 23 August 2019 and generated a new seismic sequence [53,142]. Following the final stimulation stage, trailing events included an $M_{L}$2.1 earthquake on 24 August and an $M_{L}$2.9 earthquake on 26 August [139,142]. Their occurrence after injection had ceased highlights the importance of continued monitoring following operational suspension. In November 2019, the UK government announced a moratorium on hydraulic fracturing in England following its assessment of induced-seismicity risks [144]. Although seismicity during both campaigns exhibited broadly west-to-east progression as stimulation advanced, the two wells activated different fault structures with different orientations [53,68]. These observations demonstrate that nearby wells can produce contrasting seismic responses, reflecting local differences in fault geometry and the interaction between hydraulic stimulation and pre-existing structures.

Outside the United Kingdom, the Wysin site in Poland provides another documented European example of seismicity monitored during shale-gas hydraulic fracturing. Following hydraulic-fracturing operations at Wysin in 2016, two weak seismic events were detected with estimated moment magnitudes of approximately $M_{w}1.0$ and $M_{w}0.5$ and source depths of less than 150 m. Their spatial and temporal proximity suggests a possible association with site operations, but their shallow depths and the absence of evidence for hydraulic connectivity to the injection intervals leave their origin uncertain; the smaller event may have been associated with subsequent well-site activities rather than hydraulic fracturing itself [28].

Several well-documented European cases of induced seismicity are unrelated to shale-gas development. In the Groningen field in the Netherlands, induced seismicity has been associated with conventional gas production and closely linked to reservoir compaction resulting from gas extraction [34,145]. At the Castor underground gas storage project in Spain, cushion gas injection induced a sequence of earthquakes [146]. At the Val d’Agri oilfield in Italy, water associated with hydrocarbon production has been reinjected into an unproductive marginal portion of the reservoir since June 2006, and a spatiotemporal relationship between microseismicity and wastewater injection has been observed [147]. Across Europe, shale gas development remains limited, and relatively few associated induced seismic events have been documented. The spatial distribution of representative shale-gas-related induced seismicity in Europe is shown in Figure 4.

### 3.4. China

China’s shale gas exploration and development started relatively late. Since 2005, when theoretical and technological research and development as well as exploration pilot tests were initiated, China has progressed through stages including learning-based exploration and play evaluation and selection, pilot testing, and industrial-scale development. Since 2010, major breakthroughs have been achieved in marine shale gas exploration in the Sichuan Basin and its surrounding areas, enabling large-scale and economically viable development of shale gas at depths shallower than 3500 m. These advances marked an industrial breakthrough in China’s marine shale gas sector and launched the country’s “shale gas revolution,” making China the world’s second-largest shale gas producer after the United States [148,149]. According to the EIA [135], China’s total shale gas resources are estimated at 1115.2 trillion cubic feet, accounting for about 14.7% of the global total and exceeding the combined resources of the United States and Australia. Of the nation’s technically recoverable shale gas resources, the Sichuan and Tarim basins together account for approximately 75%, with the Sichuan Basin ranking first at 56% and resources concentrated mainly in its southwestern part, followed by the Tarim Basin at about 20% [136].

By 2025, China’s commercial shale gas production was largely concentrated in the Sichuan Basin, where multiple large shale gas fields have been discovered and brought into development in areas including Fuling, Weiyuan, Zhaotong, Luzhou, Changning, Weirong, and Qijiang [150,151]. The Sichuan Basin exhibits a complex tectonic framework: its northwestern and southwestern margins adjoin the active tectonic belts of the Tibetan Plateau, and strong Cenozoic deformation and crustal movements have resulted in generally high regional seismicity characterized by relatively frequent moderate-to-strong earthquakes. Along the basin’s northwestern boundary, the Longmenshan thrust fault belt is developed and represents one of the most seismically active zones in mainland China, having hosted the 2008 Wenchuan $M_{w}7.9$ earthquake and the 2013 Ya’an $M_{w}6.9$ earthquake [152,153]. The basin’s southwestern margin is primarily controlled by the Yingjing–Mabian–Yanjin fault zone, which is dominated by left-lateral strike-slip motion; seismicity is relatively weak in its northern segment, the central segment (around Mabian County) has experienced earthquakes up to magnitude 6.8, whereas the southern segment is more seismically active, with the largest recorded earthquake being the 1974 Daguanbei M≥7 earthquake [154].

The Sichuan Basin is a typical superimposed petroliferous basin within the Tethyan tectonic domain and has long been known for its rock salt and natural gas resources [151,155]. Since 2008, the Wufeng–Longmaxi formations have become the primary target interval for intensive shale gas development in the southern Sichuan Basin, and hydraulic fracturing operations have been implemented since 2014 [156]. By 2017, more than 500 shale gas wells targeting the Wufeng–Longmaxi interval had been drilled and hydraulically fractured in this area [155]. Induced seismicity in the southwestern Sichuan Basin has been increasingly reported since the 2010 $M_{L}$5.1 Rongchang earthquake in Chongqing. Following the large-scale deployment of hydraulic fracturing techniques in 2015, shale-gas development accelerated markedly, alongside pronounced increases in both seismicity rates and event magnitudes. In recent years, multiple moderate-to-strong events have occurred in rapid succession, including the $M_{L}$5.7 Xingwen earthquake on 16 December 2018, the $M_{L}$5.3 Gongxian earthquake on 3 January 2019, and the $M_{s}$6.0 Changning earthquake on 17 June 2019. Within five days after the Changning mainshock, three additional strong aftershocks with $M_{s}>5.0$ occurred. Previous studies have linked seismicity in this region to hydraulic fracturing, water injection for salt mining, and wastewater reinjection, although the evidence and proposed mechanisms differ among earthquake sequences [18,71,155,157,158].

To date, large-scale shale gas development in the Sichuan Basin has been conducted in three demonstration areas: (1) the Changning–Xingwen block, (2) the Weiyuan-Rongchang block, and (3) the Jiaoshiba block (Figure 5). Among these demonstration blocks, the Changning block exhibits the highest level of induced seismicity. So far, ten earthquakes with M≥4.0 have been observed, including four events with M≥5.0. The Weiyuan shale gas area also shows relatively high induced seismicity, whereas the Jiaoshiba area has the lowest level of seismic activity. Following multiple tectonic episodes, the Sichuan Basin has developed several fault sets; under the present-day stress field, only a small subset of faults has relatively high slip tendency and is therefore more prone to reactivation, while a larger proportion has low slip tendency and is less favorable for rupture. This contrast may help explain why seismicity is very low in some areas (e.g., the Fuling shale gas demonstration block) but markedly higher in others (e.g., Changning and Weiyuan).

#### 3.4.1. The Changning–Xingwen Block

The Changning–Xingwen area is located on the comparatively stable southeastern margin of the Sichuan Basin along the eastern edge of the Tibetan Plateau, within a transitional zone between multiple fold belts on the southeastern side of the Huayingshan fault zone, and has historically been characterized as a region of weak and infrequent seismicity [159]. However, over the past decade it has evolved into the most concentrated area in the Sichuan Basin for moderate induced earthquakes associated with shale gas development, with more than ten events of magnitude greater than 4.0 recorded to date. Since 2018, several impactful moderate-to-strong earthquakes have occurred in this region, including the $M_{L}$5.7 Xingwen event, the $M_{L}$5.3 Gongxian event, and the $M_{s}$6.0 Changning event; the Changning mainshock was followed by a series of strong aftershocks exceeding magnitude 5.0, including the $M_{s}5.1$ Gongxian, $M_{s}$5.3 Changning, $M_{s}$5.4 Gongxian, and $M_{s}$5.6 Gongxian earthquakes.

On 16 December 2018, an $M_{L}$5.7 earthquake struck Xingwen County, Yibin City, Sichuan Province, China; two weeks later, an $M_{L}$5.3 earthquake occurred again about 8 km to the west, causing severe damage to rural housing and infrastructure in the surrounding area. Although the official focal depths for both events were reported to exceed 5 km, centroid moment tensor solutions inverted using the cut-and-paste method indicate much shallower source depths of approximately 3 km ($M_{L}$5.7) and 1.8 km ($M_{L}$5.3), respectively, and show that the epicenters were in close spatial proximity to horizontal well sections undergoing shale-gas hydraulic fracturing. Studies suggest that overpressure generated by fluid injection at depths of about 2.5~3 km triggered these moderate-magnitude earthquakes, likely by reactivating pre-existing faults driven by elevated pore pressure. In light of surface damage such as linearly distributed landslides, the source fault of the Xingwen $M_{L}$5.7 event may have nucleated within the zone influenced by fracturing and ruptured unilaterally northward for 7–8 km along a near north–south striking, steeply dipping strike-slip fault [18]. Moreover, precise relocations of the two earthquakes and their aftershock sequences, together with regional structural analysis, indicate that the frequent seismicity in this area may also be superimposed on a broader tectonic stress regime associated with southeastward extrusion of the Tibetan Plateau [155]. By contrast, the triggering factors and mechanisms of the Changning $M_{s}$6.0 earthquake sequence in June 2019 remain debated and may reflect the combined effects of shale-gas operations and brine injection for salt mining. This sequence was characterized by shallow focal depths and proximity to salt-mining districts, where long-term high-pressure solution-mining injection can enlarge salt cavities, allow fluids to migrate along pre-existing faults, and promote pressure diffusion that activates faults and generates earthquakes. This process differs markedly from the seismicity associated with the Xingwen sequence, for which the Xingwen $M_{L}$5.7 and Gongxian $M_{L}$5.3 events have been attributed to increased pore pressure on pre-existing faults caused by injection fluids migrating into the mainshock area [160,161,162].

#### 3.4.2. The Weiyuan-Rongchang Block

The Rongchang gas field is located in the border region between Sichuan and Chongqing on the southeastern margin of the Sichuan Basin. Historically, seismicity in this area was relatively low; however, since the late 1980s, with long-term reinjection of wastewater generated during natural gas production through multiple injection wells reaching depths of 2–3 km, both the frequency of earthquakes and the overall magnitude level have increased markedly. Previous studies have shown that seismicity in this region is primarily clustered around several concealed faults and exhibits a spatial pattern of outward expansion away from the injection wells, suggesting that fluid-pressure diffusion plays a key role in controlling the evolution of seismic activity [72,163].

Lei et al. [73] conducted a systematic assessment of seismicity in the Rongchang area prior to 2006 using statistical approaches including the ETAS model, and proposed stage-dependent differences in induced earthquakes that correspond to the injection history: during the early phase of injection, when injection rates were relatively low, internally driven self-triggering within the sequence dominated; during peak injection, the contribution of externally triggered, fluid-driven events increased; and as injection slowed, externally triggered seismicity weakened accordingly. Around 2010, during an intensified phase of unconventional oil and gas operations, an $M_{L}$5.1 earthquake occurred in Rongchang, with a focal depth broadly consistent with both the shale gas reservoir depth and the depth of injection operations [164]. In addition, Wang et al. [72] inverted focal mechanisms for earthquakes with $M_{L}$ ≥ 3.5 between October 2008 and July 2011 using the cut-and-paste method, further supporting the interpretation that seismicity was related to injection-induced reactivation of pre-existing faults.

With the onset of large-scale shale gas development, the Weiyuan–Rongchang area exhibited a more pronounced seismic response and an overall trend toward shallower focal depths. On 25 February 2019, an $M_{L}$4.9 earthquake occurred in Rongxian County, Sichuan Province, with a focal depth of only about 1 km, representing an extremely shallow event. Two foreshocks ($M_{L}$4.7 and $M_{L}$4.3) occurred within 2 km of the mainshock epicenter, with focal depths of ~2.7 km, comparable to the depths of hydraulic-fracturing operations. Although the cumulative Coulomb stress change contributed by the two foreshocks was only ~3 kPa, below the conventional static-triggering threshold of 10 kPa, it has been suggested that poroelastic effects may have facilitated rupture of the mainshock [165]. On September 7 of the same year, an $M_{L}$5.4 earthquake occurred in the Weiyuan area, representing the largest and most damaging induced event reported to date in the Weiyuan shale gas field. High-precision locations and migration analyses indicate that the spatiotemporal evolution of this sequence can be reasonably explained by a fluid-diffusion model with diffusion coefficients of 0.2–3.5 m^2^/s, suggesting that diffusion of high-pressure fluids within fault zones was a key triggering process. Over the subsequent four months, two additional earthquakes ($M_{L}$4.3 and $M_{L}$5.2) occurred; both had focal depths shallower than 3 km and were located within 2 km of shale gas wells. These events have been attributed primarily to continued fluid diffusion and elevated pore pressure, rather than solely to static stress transfer from the $M_{L}$5.4 event [166]. However, other studies have argued that the $M_{L}$5.2 earthquake on 18 December 2019 occurred within a region where the preceding $M_{L}$5.4 earthquake had increased Coulomb stress by approximately 10 kPa, reaching the commonly adopted static-triggering threshold; thus, in addition to fluid-diffusion effects, stress perturbations imparted by prior ruptures may also have contributed to seismicity in the Weiyuan–Rongxian area [167]. Further fault-reactivation analyses suggest that the incremental fluid pressure required to bring faults to a critical state of instability may be as low as 1.3–10.6 MPa [17], underscoring that seismicity in this region likely reflects the coupled influence of tectonic conditions and fluid injection.

#### 3.4.3. The Jiaoshiba Block

The Jiaoshiba block of the Fuling shale gas field in Chongqing is located in the steep fold-and-thrust belt in the eastern Sichuan Basin, and its boundaries are controlled by multiple faults. It can be subdivided into two major structural belts: the NE-trending Jiaoshiba faulted anticline and the NW-trending Wujiang faulted anticline [168]. In late 2012, Sinopec achieved a major breakthrough with the Jiao Ye 1HF well targeting the organic-rich shale interval spanning the Upper Ordovician Wufeng Formation and the Lower Silurian Longmaxi Formation. By the end of 2015, productivity testing had been completed for more than 190 wells, proven geological reserves reached $3805.98\times 10^{8}$ m^3^, and the constructed production capacity reached $50\times 10^{8}$ m^3^.

Based on structural interpretation combined with extensive well-test data from horizontal wells, studies generally suggest that shale-gas well productivity in the Jiaoshiba block is strongly heterogeneous in space and that the dominant controlling factors vary across structural units. Specifically, in units with well-developed fault structures, the geometry and kinematic characteristics of major faults often govern stimulation effectiveness and well productivity. In units where fault influence is relatively limited, however, variations in productivity are better explained by the density and connectivity of natural fracture networks, underscoring the key role of fracture systems in regulating reservoir permeability and flow capacity [168]. In addition, to further clarify the relationship between tectonic activity and microseismicity, portable seismic arrays have been deployed for enhanced monitoring. Observations indicate that microseismic events cluster along faults near the production-area boundaries, with a pronounced concentration along the southeastern peripheral fault zone, and hypocentral depths predominantly between 2 and 6 km. Current interpretations tend to link this microseismicity to local stress perturbations associated with engineering operations and the slip propensity of boundary faults, whereas regional-scale seismicity remains constrained by the background regional stress field. Overall, reported microseismic events in the Jiaoshiba block have been small in magnitude, with limited documented impacts [169].

### 3.5. Regional Variations in Induced Seismicity and Controlling Factors

Regional differences in seismic response need to be understood within their operational and geological settings. The central United States sequences discussed above are mainly associated with prolonged wastewater disposal, whereas the Canadian and UK cases show clearer spatiotemporal links to hydraulic fracturing [8,10,12,53]. The Sichuan Basin hosts hydraulic fracturing, wastewater disposal and water injection for salt mining, with studies linking individual sequences to different activities [17,76,170]. Regional comparisons, therefore, require more than identifying the main operation type; they must also examine how particular operations affect local faults.

These effects begin with how perturbations are applied and how long the injection continues. Some wastewater disposal areas in the central United States involve prolonged injection, large cumulative volumes and multiple operating wells. Pressure perturbations can consequently propagate outwards and affect faults distant from disposal wells [8,10]. Hydraulic fracturing typically involves brief, high-pressure, staged injection, with seismic responses often closely linked to individual stages and treatment schedules [12,53]. However, a brief injection at an individual stage does not imply that perturbations remain nearby or disappear when treatment ends. Their subsequent evolution also depends on hydraulic connectivity within formations and fault systems [12,171]. Hydraulic connectivity thus links operational processes to fault responses. Studies in Oklahoma indicate that connections between disposal formations and basement faults allow prolonged injection to influence deeper seismicity [8,10]. Dense monitoring in Alberta further shows that pre-existing fracture corridors can transmit pressure perturbations from hydraulic fracturing to larger faults [171]. At Preston New Road, stress transfer caused by fracture opening may also have promoted fault slip [53]. Pressure diffusion and stress transfer should therefore not be treated as mechanisms exclusive to wastewater disposal and hydraulic fracturing, respectively. Their relative contributions depend on fault orientation, initial stress and connections to the injection interval [5,12].

These fault conditions influence both earthquake occurrence and the extent of rupture. Representative cases include the $M_{w}$5.8 Pawnee earthquake [57], the $M_{w}$4.6 Fort St. John earthquake [51] and the $M_{L}$2.9 Preston New Road event [53]. Reports from Sichuan include an $\;M_{L}$5.7 event associated with hydraulic fracturing and the $M_{s}$5.4 Changning earthquake potentially linked to salt-mining injection [18,76]. These values represent different cases and magnitude scales, so they cannot directly establish differences in magnitude limits between operation types. Rupture modeling of the Pawnee earthquake also suggests that rupture extended beyond the zone of stronger pore-pressure perturbation [172]. The extent of the perturbation therefore need not coincide with the final rupture area.

Similarly, the end of operations does not necessarily mark the end of fault response. Cases in western Canada and the United Kingdom document seismicity after injection ceased, showing that hazard assessments must extend into the post-injection period [12,15,53]. Delayed occurrence alone, however, cannot distinguish the effects of pressure diffusion, stress adjustment and earthquake nucleation [5]. Regional assessments should therefore consider injection rate, cumulative volume, operational duration, hydraulic connectivity, initial stress and potential rupture dimensions. Differences in monitoring capabilities and observation periods also require consideration. Where activities coexist or operational records are incomplete, multiple possible sources should be distinguished from demonstrated combined effects, with attribution uncertainty explicitly retained.

## 4. Forecasting, Geospatial Susceptibility Assessment, and Mitigation of Shale-Gas-Induced Seismicity

### 4.1. Forecasting Targets and Modeling Approaches

Induced-seismicity assessment involves distinct targets, including maximum magnitude, seismicity rate, spatial susceptibility, and operational risk. Maximum-magnitude assessment may constrain the largest event under a specified injection scenario and explicit physical assumptions [23]. Alternatively, probabilistic models estimate the likelihood of exceeding a specified magnitude during active injection [173]. Seismicity-rate forecasting estimates the expected number of earthquakes above a specified magnitude threshold per unit time [21]. Spatial susceptibility assessment identifies locations where geological conditions favour induced seismicity in the context of injection operations, without necessarily specifying event timing [129]. Operational risk assessment combines seismic hazard with exposure and vulnerability to evaluate potential consequences and inform operational decisions [174]. Here, we distinguish these assessment targets from statistical, physics-based, hybrid, and machine-learning approaches, which describe how an assessment is performed. These approaches do not correspond one-to-one with assessment targets: a single approach may address multiple targets, and different approaches may address the same target, as illustrated by statistical magnitude-probability models, coupled physical–statistical forecasts, and machine-learning susceptibility assessments [21,129,173].

Among these targets, maximum-magnitude estimation is particularly important because larger induced earthquakes can threaten safety, affect public acceptance, and trigger regulatory restrictions on continued operations. Previous studies have documented earthquakes of magnitude 3 or greater associated with industrial activities. In many cases, their focal mechanisms resemble those of natural tectonic earthquakes. However, a systematic understanding of the physical causes of induced seismicity and its spatiotemporal evolution has only begun to make substantive progress in recent years. On the one hand, the time-varying nature of operational parameters (e.g., adjustments in injection rate, wellhead pressure, and stage transitions) introduces pronounced temporal nonstationarity, leading to stage-dependent fluctuations in seismicity rates throughout operations. On the other hand, heterogeneity in regional geological conditions (e.g., the initial tectonic stress field, fault geometry and connectivity, and formation permeability) further results in markedly different seismic responses across blocks under comparable injected volumes and operating conditions: some areas exhibit intensified seismicity accompanied by an upward shift in magnitudes, whereas others remain relatively quiescent. Overall, induced seismicity is jointly governed by multiple natural and engineering factors and therefore exhibits strong spatiotemporal complexity and regional variability [68,173,175]. Nevertheless, compared with the inherently higher uncertainty of natural earthquakes [47], injection-induced seismicity is commonly regarded as more readily constrainable for forecasting purposes, largely because industrial operations are, to some extent, controllable and because relatively rich physical parameters and real-time downhole monitoring data can be obtained during field operations.

Current understanding of the physical mechanisms underlying induced seismicity during shale oil and gas development encompasses static Coulomb stress changes, pore-fluid pressure diffusion, poroelastic responses of the medium, and aseismic slip [70,176]. Building on these mechanistic insights, research on induced seismicity in shale oil and gas development has focused particularly on approaches to forecasting the maximum expected magnitude, and three broad methodological paradigms have emerged [176,177] (Figure 6). The first comprises statistical-based approaches, which typically leverage earthquake catalogs and apply statistical tools such as the epidemic-type aftershock sequence (ETAS) model to characterize the spatiotemporal distribution of background and induced events, thereby inferring seismicity rates [174,178]. The second comprises physics-based approaches that emphasize numerical simulations to capture fluid migration and pressure evolution during injection, as well as the resulting stress-field changes and fault-instability conditions; these methods generally require well-constrained geological and operational parameters, including the geometries of reservoirs and faults, initial pore pressure and in-situ stress states, permeability, and temperature [142,179]. The third comprises hybrid modeling approaches, in which physical simulations are first used to track the system’s evolution toward instability, and statistical methods are then applied to characterize the seismicity following instability, aiming to balance physical realism with statistical predictive capability [177,179]. In recent years, artificial intelligence (AI) approaches have also been introduced into induced-seismicity research, using machine learning and deep learning to automatically extract features from large volumes of seismic monitoring data, identify anomalous patterns, and forecast trends in seismic activity, thereby offering potential advantages in short-term prediction accuracy and real-time early-warning performance [161,180].

#### 4.1.1. Statistical Approaches

Statistical models typically take seismic catalogs from the pre-operational and operational periods as their primary data source and characterize the temporal evolution of induced seismicity to provide real-time or near-real-time forecasts of event occurrence probabilities and potential maximum magnitudes under constraints imposed by a small number of calibratable parameters. These approaches share several common features: they adopt relatively simple model structures, entail low computational cost, and rely mainly on the statistical regularities of seismic catalogs as the principal information carrier, while treating physical processes such as fluid migration, pore-pressure evolution, and frictional fault instability only implicitly or through approximate parameterizations. Consequently, statistical frameworks have been widely adopted in engineering risk-management settings, particularly for rapid updating and operational decision support [21,24,173,182,183,184,185].

Existing studies generally fall into two categories. The first category combines the Gutenberg–Richter (G-R) relationship with finite-sample extreme-value theory, estimating the most probable sample maximum of an unbounded G–R distribution for a finite number of events. Van der Elst et al. [24] showed that, given the magnitude of completeness $M_{c}$ and the *b*-value, if *N* denotes the number of events with $M\geq M_{c}$, the most probable maximum magnitude can be expressed as $M_{max}=M_{c}+\frac{1}{b}{log}_{10}N$. If induced earthquakes are further assumed to occur predominantly on pre-existing faults, then the potential maximum magnitude can, in a statistical sense, be regarded as not exceeding the largest rupture size permitted by the local fault system, implying that it may be comparable to, or share a similar upper-bound constraint with, the maximum magnitude of natural earthquakes in the same region. However, this inference is strongly dependent on sample size, catalogue completeness, and the regional tectonic setting, and therefore requires site-specific validation.

The second category constrains induced seismicity using either injected volume or seismic-moment conservation, emphasizing that the cumulative moment release of an induced sequence is subject to an estimable upper bound or follows a scaling relation. The idea was originally proposed in the context of subsurface extraction, where production-induced volume change could cap the maximum seismic moment release, and was later extended to injection-induced seismicity in the form of a simplified expression in which the net injected volume controls the upper bound of moment release [23]. For a medium with shear modulus *μ*, the upper bound on the seismic moment released by induced earthquakes can be approximated as $M_{0}^{max}=\mu ∆V$, where ∆V is the net injected fluid volume. This estimate implicitly assumes that the medium is approximately fully saturated and that the fault system is near a critical failure state, such that even modest pressure increases can promote fault slip. It should be emphasized that this approach provides a theoretical upper bound rather than a magnitude that must be achieved. Most hydraulically induced events have magnitudes well below this bound; however, in regions such as the Western Canada Sedimentary Basin (WCSB), events exceeding the bound have been reported, suggesting that the underlying assumptions may be violated at certain sites or that additional mechanisms may be involved [11,133]. Building on this framework, Li et al. [186] proposed that continuously tracking the temporal evolution of cumulative seismic moment can enhance the sensitivity and updatability of short-term forecasting, reflecting a shift from static caps toward dynamic constraints. In practice, volume- or moment-based models typically follow a similar workflow: a scaling relation between ∆V and $\Sigma M_{0}$ is established early during injection and extrapolated to estimate the expected total moment release over the full injection period; given an estimated *b*-value, the expected maximum magnitude and its uncertainty range can then be derived. In addition, to capture post-injection trailing activity, some studies incorporate a term analogous to aftershock decay in event-rate models, thereby better reproducing the commonly observed phenomenon of sustained activity after shut-in [174,187]. Nevertheless, available evidence indicates that the scaling between ∆V and seismicity is often not stationary during operations and may drift with injection strategy, evolving formation permeability, fault connectivity, and changes in stress conditions. Such non-stationarity limits the portability and robustness of these approaches for constraining maximum magnitude and supporting prospective risk management, and practical applications remain in an exploratory stage.

In addition to the two categories above, some studies employ empirical or geometrically constrained relationships to bound the maximum rupture dimension. Shapiro et al. [173], for instance, proposed a geometry-based approach that assumes induced ruptures are fully contained within the stimulated zone, such that the maximum magnitude is controlled by the size of the stimulated region. Recent observations, however, show that rupture surfaces can extend ~2 km beyond the stimulated zone [12], indicating that the maximum rupture size is not necessarily limited by the spatial footprint of the observed microseismic cloud. Overall, statistical models retain notable conceptual and computational advantages: they can extrapolate the future distribution of events at relatively low cost when detailed physical parameters are unavailable and typically provide a statistical estimate of the maximum magnitude, which underpins their widespread use in induced-seismicity studies and operational risk management.

It should be noted that statistical estimates of maximum magnitude depend not only on model formulation, but also on the characteristics of seismicity associated with different injection practices. As an empirical complement to the statistical constraints discussed above, Figure 7 compares the distributions of *b*-value and maximum magnitude for induced seismicity associated with hydraulic fracturing and wastewater disposal. Overall, wastewater disposal cases are characterized by higher maximum magnitudes and a broader range of variation, whereas hydraulic fracturing cases are more concentrated in the lower magnitude range. Although the two categories overlap to some extent in *b*-value, the contrast is more evident along the $M_{max}$ axis. This difference likely reflects the distinct ways in which the two activities perturb subsurface fault systems. Hydraulic fracturing is typically a short-term, spatially localized, stage-based injection process, and the resulting seismicity is commonly associated with small-scale fracture propagation and localized fault slip within or near the target reservoir, thereby favoring microseismicity and small events. In contrast, wastewater disposal generally involves longer injection durations, larger cumulative fluid volumes, and a broader extent of fluid migration, making it more likely to reactivate deeper preexisting faults through pore pressure diffusion and stress transfer, particularly basement faults that are already close to failure, and thus increasing the likelihood of moderate to larger induced earthquakes. Moreover, the *b*-value primarily describes the frequency–magnitude distribution and is not directly linked to maximum magnitude. Its estimation is also sensitive to the magnitude of completeness ($M_{C}$), monitoring capability, and the time window adopted for analysis. The overlap between the two categories in *b*-value is therefore not unexpected. Taken together, the observed separation should not be interpreted as a simple binary distinction, but rather as the combined effect of injection mode, fault architecture, regional stress conditions, and data selection.

**Figure 7:**
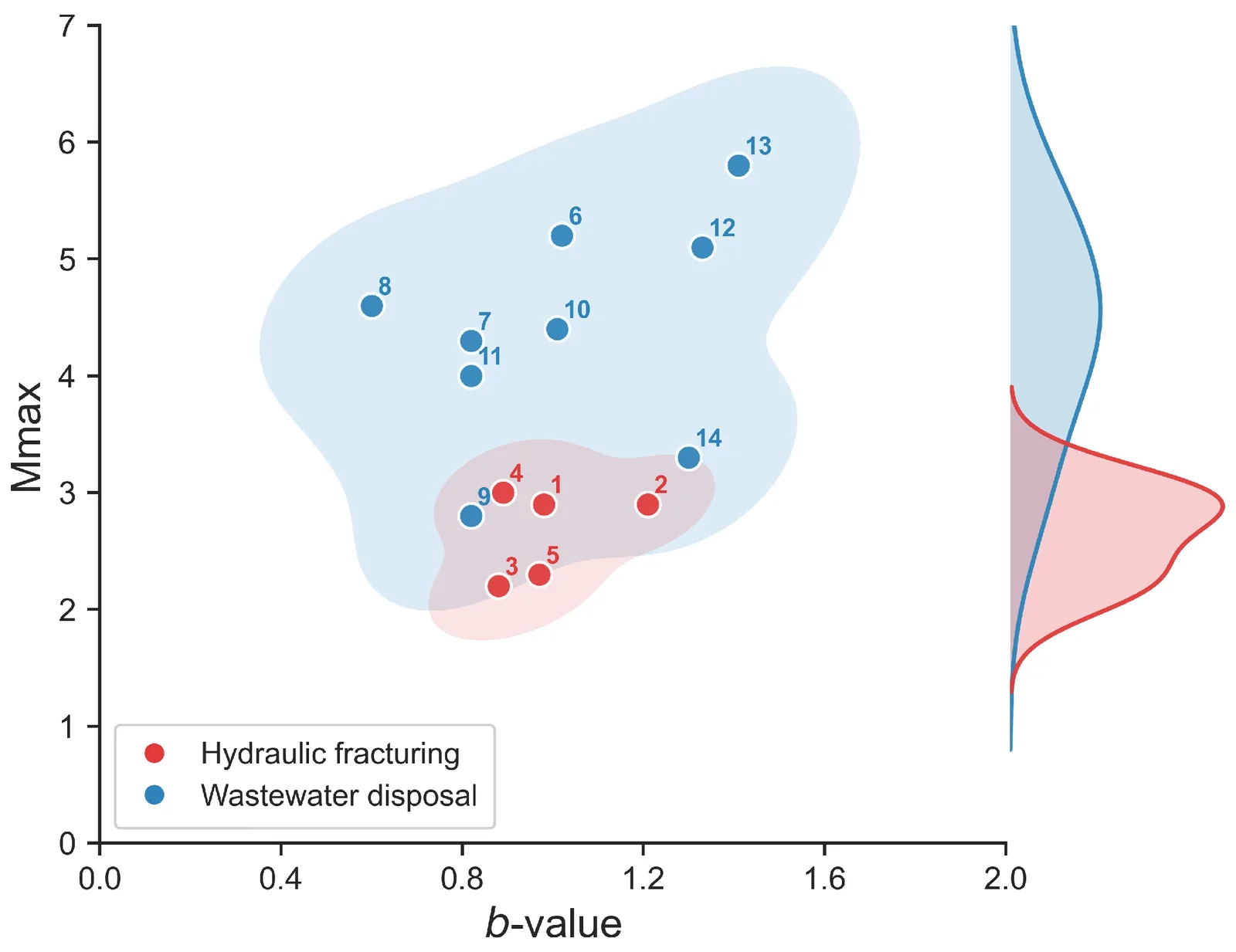
Relationship between reported *b*-values and maximum magnitudes for hydraulic-fracturing- and wastewater-disposal-related induced seismicity sequences. Points represent individual sequences listed in Table 3.

**Table 3:** Representative hydraulic-fracturing- and wastewater-disposal-related induced seismicity sequences with reported maximum magnitudes and *b*-values.

Operation Type	No.	Date	Location	$M_{max}$	*b*-Value	References
HF	1	18 January 2011	South-central OK, USA	$M_{L}$2.9	0.98	[65]
	2	19 May 2011	Horn River Basin, BC, Canada	$M_{L}$2.9	1.21	[114]
	3	October 2013	Harrison County, OH, USA	$M_{w}$2.2	0.88	[86]
	4	11 March 2014	Poland Township, OH, USA	$M_{L}$3.0	0.89	[188]
	5	6 April 2023	Southern Sichuan Basin, China	M2.3	0.97	[74]
WD	6	13 August 1997	Rongchang District, Chongqing, China	$M_{L}$5.2	1.02	[73]
	7	May 2005	Paradox Valley, CO, USA	M4.3	0.82	[189]
	8	22 September 2005	Southern Central Valley, CA, USA	$M_{w}$4.6	0.60	[190]
	9	9 June 2009	Cleburne, TX, USA	$M_{L}$2.8	0.82	[191]
	10	27 February 2011	Guy–Greenbrier, AR, USA	$M_{L}$4.4	1.01	[96]
	11	31 December 2011	Youngstown, OH, USA	M4.0	0.82	[192]
	12	13 February 2016	Fairview, OK, USA	$M_{w}$5.1	1.33	[22]
	13	3 September 2016	Pawnee, OK, USA	$M_{w}$5.8	1.41	
	14	16 May 2009	Dallas–Fort Worth, TX, USA	M3.3	1.30	[59]

Although b-value and $M_{\max}$ provide useful measures of earthquake-size distributions, they do not fully characterize changes in the temporal clustering, spatial migration, and statistical organization of seismicity during injection. Information-theoretic approaches provide a complementary perspective by quantifying the statistical organization and complexity of earthquake sequences [193,194]. In particular, entropy measures derived from inter-event times or spatial distributions may help identify changes in seismic organization that are not readily reflected by conventional seismicity rates or *b*-value. Applied to induced seismicity, it could therefore be used to test whether operational parameters, such as injection rate, cumulative injected volume, and wellhead pressure, provide information about subsequent seismic activity beyond that contained in the earthquake history itself. The principal value of these measures lies not in directly predicting $M_{max}$, but in identifying changes in seismic states, including transitions from spatially dispersed activity to persistent clustering, directional migration, or concentration along specific fault structures. Nevertheless, entropy-based measures are sensitive to catalogue completeness, analysis-window selection, monitoring capability, and time-series length, while their direct application to injection-induced seismicity remains limited. At present, they are therefore best regarded as complementary diagnostic tools for linking earthquake-catalogue statistics with injection processes and geomechanical responses, rather than as standalone forecasting metrics.

#### 4.1.2. Physics-Based Approaches

Physics-based approaches simulate injection-induced changes in pore pressure and stress and evaluate their effects on fault stability using mechanical failure criteria. These models represent subsurface conditions through geological structure, hydraulic and mechanical properties, and the initial stress state, with injection histories imposed as operational forcing. Depending on the operational setting and modelling objectives, the physical processes considered may include pore-pressure diffusion, poroelastic coupling, thermoelastic stresses, and aseismic slip [176].

From a mechanistic perspective, Johann et al. [195] systematically incorporated three coupled processes, including injection-induced changes in normal stress acting on basement faults, pore-pressure diffusion, and poroelastic modulation of the stress field. They further suggested that reservoir-type induced seismicity is more likely to occur in tectonic settings dominated by normal and strike-slip faulting, with the strike-slip regime in Oklahoma serving as a representative example. This framework not only provides a physical basis for the sensitivity of seismicity to injection volume and injection depth, but also supports more physically constrained approaches to induced-seismicity forecasting, mitigation strategy development, and disposal-site selection. Such physics-based models do not rely primarily on empirical extrapolation from past earthquake sequences; instead, they leverage initial conditions including subsurface structure, fault geometry, and injection parameters to simulate the evolution of pore pressure, stress perturbations, and fault stability, thereby providing a mechanistic basis for prospective assessment of induced-seismicity potential prior to the onset of injection operations.

#### 4.1.3. Hybrid Approaches

Hybrid forecasting approaches, which integrate physics-based and statistical models, have demonstrated clear advantages in induced-seismicity prediction by combining complementary strengths and compensating for the limitations of each component. In general, current hybrid frameworks employ geomechanical models to quantitatively simulate stress perturbations associated with shale-gas development, particularly large-scale hydraulic fracturing, and to formulate fault-instability criteria. These physics-based constraints are then coupled with statistical models to estimate the probabilistic distribution of potential seismic energy release, supporting seismicity forecasts that are both physically grounded and statistically adaptive.

A representative class of hybrid models couples poroelastic or hydromechanical simulations with rate-and-state formulations of seismicity. For wastewater injection in western Oklahoma and southern Kansas between 2010 and 2018, a linear poroelastic model was used to calculate pore-pressure diffusion and poroelastic stress changes. These physically derived perturbations were then incorporated into a rate-and-state model to estimate the temporal evolution of seismicity rates. The results showed that injection-related perturbations could propagate far beyond the immediate injection zone. Injection in Oklahoma increased Coulomb stress changes in Kansas by approximately 1.5-fold and was associated with up to a threefold increase in seismicity rates [196]. Similarly, a reservoir-scale model incorporating more than two decades of injection records from 902 saltwater-disposal wells in the Arbuckle aquifer was used to calculate stressing-rate changes on basement faults. These changes were subsequently introduced into a rate-and-state seismicity model. The coupled framework reproduced the observed onset, peak, and subsequent decline of seismicity after injection reductions. It also captured the time lag between changes in injection and the corresponding seismicity-rate response [197]. Zhai et al. [25] likewise combined linear poroelastic calculations of injection-induced crustal stress perturbations with a rate-and-state formulation to estimate seismicity evolution under different fault orientations. Together, these studies illustrate a common hybrid architecture in which physically calculated pressure or stress perturbations provide the forcing variables for statistical models of earthquake occurrence.

Other hybrid approaches use alternative statistical formulations to translate physically modelled perturbations into seismicity forecasts. Models that incorporate pore-pressure evolution, basement-fault distributions, and spatially variable stress conditions have been developed to forecast the spatiotemporal probability of damaging induced earthquakes in Oklahoma and Kansas [21]. In the Raton Basin of Colorado and New Mexico, a three-dimensional finite-element poroelastic model was used to calculate changes in Coulomb stress associated with more than two decades of wastewater injection. The resulting stress rates were then coupled with a spatially calibrated seismogenic index model to forecast the spatiotemporal evolution of induced seismicity [179]. Fluid-flow simulations have also been used to modulate background seismicity rates within ETAS-type models, thereby improving the representation of frequency–magnitude distributions and the estimation of larger-event probabilities [198]. Collectively, these studies demonstrate that hybrid forecasting does not represent a single model architecture. Instead, it encompasses a range of frameworks that quantitatively link physically derived state variables or stress perturbations to statistical descriptions of earthquake occurrence. Such coupling preserves mechanistic information while enabling probabilistic forecasts that can be updated dynamically as new monitoring data become available.

#### 4.1.4. Machine-Learning Approaches

With improvements in monitoring resolution and the expansion of available datasets, AI methods have increasingly been applied to extract seismic features and forecast seismicity trends, providing new technical support for seismic-risk management associated with energy-development activities such as shale-gas production and wastewater injection. However, the literature widely recognizes that these approaches still face major bottlenecks in cross-region generalization and physical interpretability, underscoring the need to integrate them with physics-based and statistical models to achieve more reliable forecasting. In recent years, a range of machine-learning algorithms has been used to quantify key controls on induced seismicity. For example, a Random Forest (RF) model that integrates injection parameters (rate and pressure), geological information, and simulated poroelastic stresses successfully predicted induced seismicity rates in Oklahoma (R^2^ = 0.75), indicating a strong association between operational parameters and seismic activity [199]. In Canada’s Western Canadian Sedimentary Basin, machine-learning models have demonstrated the ability to identify susceptibility to induced seismicity across multiple spatial scales. At the regional scale, models constructed from tectonic context, in situ stress conditions, and hydrological variables can identify susceptibility to induced seismicity and delineate areas of relatively high susceptibility. At the operational scale, models that combine hydraulic-fracturing operational data with geological factors can reconstruct the spatial distribution of induced events with high fidelity and inform parameter adjustments to mitigate induced seismicity [46,129]. Notably, the spatiotemporal distribution of induced seismicity is influenced not only by operational parameters but also by natural background conditions including stratigraphic architecture, fault attributes, and hydrogeological settings, and the dominant controls may differ substantially among regions. For instance, in the Northeast Montney Play in British Columbia, Canada, an XGBoost model coupled with SHAP analysis indicated that injection parameters contribute more prominently to seismic activity, whereas formation thickness and fault density exert relatively weaker effects [20]. By contrast, seismicity in the Southern Montney region is more strongly controlled by the number of fracturing stages, cumulative historical injection volumes, and structural position [200], further highlighting the influence of regional geological heterogeneity on model transferability and interpretation.

To enhance the physical interpretability of forecasting, recent research has explored strategies that embed machine learning within physics-based process models or couple machine-learning components with physical models. For example, in Colorado’s Paradox Valley, a poroelastic stress model combined with a machine-learning classifier successfully distinguished injection-driven events from earthquake-driven events, indicating that approximately 22% of the earthquakes were directly triggered by injection, whereas the remainder likely represented aftershock sequences [179,201]. In addition, some studies have integrated numerical simulations of fluid flow and dynamic rupture propagation with machine-learning regression and Bayesian inversion of subsurface model parameters; using the 2012 Texas M4.8 earthquake as a case study, they supported a mechanism whereby wastewater injection triggered fault slip through poroelastic stressing [202]. Machine learning has also been applied to refine the basic framework of adaptive traffic-light systems by training surrogate models to capture the nonlinear relationships between threshold magnitude and operational parameters, thereby improving both adaptability and practical implementability [203]. Overall, machine learning holds substantial promise for identifying key controlling factors, enhancing spatial forecasting, and supporting risk management; however, its transferability and interpretability still need to be strengthened through deeper integration with physical constraints and statistical models.

Across the approaches reviewed above, regional observations inform model inputs, parameters, and state variables. Fault geometry, permeability, and initial stress constrain model configuration, whereas injection histories provide operational forcing. Physical models simulate the evolution of pore pressure and fault stress, while statistical and machine-learning models use operational and geological factors as predictors. Seismic observations support calibration and updating, with monitoring coverage and catalog completeness affecting forecast uncertainty. Table 4 summarizes representative models, their predictor variables, core relationships, and primary outputs.

### 4.2. Geospatial Susceptibility Mapping for Operational Decision Support

Maximum-magnitude forecasting constrains how large an induced event may become, whereas geospatial susceptibility mapping identifies where induced seismicity is relatively more likely to occur. Geospatial susceptibility mapping, therefore, provides a spatial extension of induced-seismicity forecasting by integrating geological, seismological, and operational variables into a spatially explicit susceptibility framework [21,204]. Candidate factors include the distance between injection intervals and mapped or inferred faults, proximity to crystalline basement, fault architecture and hydraulic connectivity, fault stability under the present-day stress field, reservoir hydraulic properties, historical seismicity, cumulative injection volume, injection rate, and wellhead pressure [9,21,44,91,176]. Monitoring-network coverage and detection capability should additionally be considered when assessing catalogue completeness and the uncertainty of susceptibility estimates, rather than being treated as direct controls on the physical susceptibility of the subsurface [174,204]. The geological and operational factors described above represent different components of the induced-seismicity system, including structural predisposition to failure, hydraulic and poroelastic stress perturbations, and operational forcing [21,44,176]. Their relevance and predictive value are site- and scale-dependent and should be evaluated jointly rather than assumed to be universal [9,21,91]. When the assessment moves from seismic hazard to seismic risk, exposure and vulnerability indicators, such as population density, critical infrastructure, and building vulnerability, should also be incorporated [26,44].

This spatial perspective is particularly important because damaging induced earthquakes can involve the reactivation of subsurface faults that are incompletely characterized before operations [204,205]. Susceptibility maps should therefore be treated as dynamic decision-support layers rather than a static product [21,204]. Building on time-dependent forecasting and monitoring-informed operational management, susceptibility assessments can be periodically revised as new seismic, operational, and subsurface information becomes available, including newly detected seismicity, updated injection records, improved hypocentral locations, focal-mechanism solutions, and geomechanical-model outputs [21,204,206,207]. Conversely, susceptibility estimates in areas with limited monitoring capability should be interpreted cautiously because catalogue incompleteness may obscure or delay recognition of emerging seismic activity [174,204]. In this sense, geospatial susceptibility assessment provides a structured basis for identifying where additional characterization, monitoring, and operational control may be most warranted, thereby linking mechanistic understanding with operational decision-making.

### 4.3. Operational Monitoring and Risk-Control Measures

Because critically stressed pre-existing faults are widely regarded as a prerequisite for hydraulic-fracturing-induced earthquakes, maintaining a minimum offset, or “safe distance”, between fracturing operations and such faults has been proposed as a preventive measure [208]. Its practical effectiveness is limited, however, by uncertainty in subsurface fault detection and characterization. Strike-slip faults can be particularly difficult to identify before reactivation, and structures mapped by pre-injection geophysical surveys do not always correspond clearly to those that subsequently host seismicity [141,209]. In some documented cases, the causative fault has been more clearly characterized only after the occurrence of an earthquake sequence [13,14].

Dedicated seismic monitoring provides the observational basis for detecting and characterizing induced seismicity and for implementing timely operational response. Rather than merely documenting earthquakes after they occur, monitoring systems should be designed to achieve an appropriate magnitude of completeness and location accuracy, detect changes in seismicity, and provide sufficiently reliable source parameters for operational decision-making [3,204]. The USGS and its academic partners have deployed monitoring instruments at known or suspected sites of injection-induced seismicity across several central and eastern U.S. states [210]. Comparable local monitoring networks have been established in shale-gas-producing areas of the Sichuan Basin to improve the detection and location of low-magnitude seismicity [19,170]. In the Montney play of British Columbia, dense local arrays combining geophones and accelerographs have been deployed to monitor induced seismicity [211]. Within designated monitoring and mitigation areas, regulatory requirements combine pre-operational assessment, real-time seismic monitoring, and event-triggered operational responses [212]. Because risk-control decisions often depend on earthquake magnitude and peak ground velocity, monitoring networks may require both velocity seismometers and strong-motion accelerometers. Borehole instruments can further improve event detection and hypocentral-location accuracy [3,211]. The adoption of dedicated monitoring standards in several countries highlights the regulatory importance of network performance [44,204,212,213]. Monitoring capability should therefore be explicitly considered when evaluating the reliability of seismicity characterization and the effectiveness of operational response systems.

The observations generated by these monitoring systems acquire operational value when they are linked to predefined, tiered response mechanisms, most commonly implemented through traffic-light systems (TLSs). Conventional TLSs link predefined decision variables, such as earthquake magnitude or ground-motion measures, to green, amber, and red operational response levels. Under a conventional three-level framework, green generally permits normal operation, amber triggers enhanced monitoring and/or mitigation measures such as adjustment of injection parameters, and red requires suspension or shut-in of operations [3,204]. Although conventional TLSs provide transparent and readily implementable decision rules, fixed thresholds may not adequately account for site-specific differences in seismic hazard, exposure, vulnerability, or the possibility of continued seismicity after injection is reduced or suspended [26,204].

Risk-based approaches address some of these limitations by deriving operational thresholds from acceptable levels of seismic impact or risk rather than applying a uniform magnitude threshold across an entire region [26,204,214]. For example, risk-informed red-light thresholds can incorporate the forecast maximum magnitude, source depth, site amplification, ground-motion relationships, tolerances for nuisance or damaging shaking, and population exposure, allowing threshold magnitudes to vary spatially according to the consequences that an induced event may produce [26,214]. Adaptive TLSs provide a complementary development by updating hazard or risk estimates and associated operational responses as new seismic and operational observations become available, rather than relying exclusively on conditions fixed before operations [174,204]. By incorporating the evolving seismic response to ongoing injection, such systems can provide a more dynamic basis for operational decision-making; however, their effectiveness depends on sufficiently sensitive real-time monitoring, reliable forecasting models, and clearly defined risk or safety criteria [174,204].

At the PNR-1z well in the Lancashire shale-gas project in the United Kingdom, six events exceeded the TLS thresholds after approximately two weeks of operation in 2018. Following the implementation of risk-control measures, activities were suspended for about one month, and no subsequent events exceeded the red-light threshold [143]. In Alberta, Canada, a shale-gas project experienced two red-light events in June 2015 and January 2016. Hydraulic fracturing was halted after each event and reported to the Alberta Energy Regulator (AER) within three hours. Operations subsequently resumed after regulatory review, and no further earthquakes exceeding the red threshold were observed following resumption [215]. In Oklahoma, the revised well-completion seismicity protocol, issued in February 2018, required operators to monitor seismicity within 5 km of hydraulic fracturing operations and to implement magnitude-dependent responses. Events with $2.0\leq M_{L}<2.5$ triggered the operator’s seismicity response plan, while events with $2.5\leq M_{L}<3.0$ required notification and, where applicable, a six-hour operational pause. Events with $3.0\leq M_{L}<3.5$ required a six-hour pause and technical consultation, whereas events with $M_{L}\geq 3.5$ required immediate suspension and an in-person technical conference [216]. Induced seismicity in Oklahoma has declined in recent years under this broader control environment, although the contribution of individual measures requires cautious interpretation. Collectively, these applications illustrate the operational value of tiered response systems, but also reveal their dependence on threshold design, catalogue completeness, enforcement speed and the capacity to account for delayed or post-injection seismicity.

Technical monitoring and operational thresholds must consequently be embedded within a wider regulatory framework. Historically, shale-gas development in England involved petroleum licensing, local planning permission, environmental permitting, and well-safety oversight. Operators were required to notify the Health and Safety Executive of well design and operational plans at least 21 days before drilling, while hydraulic fracturing plans addressed induced seismicity risks, as well as monitoring and response procedures. Community engagement was also supported by voluntary industry commitments, including £100,000 in community benefits per well-site where hydraulic fracturing took place at the exploration stage [217,218]. Selected regulatory measures for shale-gas-related induced seismicity are summarized in Table 5.

Other jurisdictions have developed different combinations of monitoring standards, operational response requirements, and public communication mechanisms. In China, Chongqing introduced a recommended local standard for seismic monitoring in shale-gas development areas [213]. In British Columbia, designated seismic monitoring and mitigation areas combine pre-operational assessments, real-time monitoring, and event-triggered operational responses [212]. These examples highlight the importance of clearly specified monitoring objectives, operational responsibilities, and response procedures in translating seismic observations into risk-management decisions.

## 5. Summary and Discussion

Shale-gas-related induced seismicity is governed not by injection volume alone, but by the interaction between operational forcing and the pre-existing geomechanical state of the subsurface. Evidence from the central United States, western Canada, the United Kingdom and the Sichuan Basin shows that similar operations can produce markedly different seismic responses. The potential for larger induced earthquakes therefore depends on whether injection perturbs critically stressed faults that are hydraulically or mechanically connected to the injection zone.

This perspective also qualifies the conventional distinction between hydraulic fracturing and wastewater disposal. Hydraulic fracturing is generally short-lived, stage-based and spatially localized, and thus commonly produces near-reservoir microseismicity. Wastewater disposal typically continues for longer and involves larger cumulative volumes, allowing pore-pressure diffusion and poroelastic stress transfer to perturb broader fault systems. These differences reflect contrasting forcing styles, however, rather than a fixed difference in seismic hazard. Either activity may induce moderate earthquakes when perturbations reach favourably oriented and sufficiently stressed faults. Attribution is particularly difficult in structurally complex regions such as the Sichuan Basin, where hydraulic fracturing, wastewater injection, solution mining, dense fault networks and regional tectonic loading coexist. Operational metrics therefore acquire predictive value only when interpreted within their geological and geomechanical settings.

The strong dependence on poorly characterized fault conditions helps explain why induced-seismicity forecasting remains uncertain. Microseismicity near well sites can reveal local fracture growth, whereas the delayed activation of distant or blind faults is much more difficult to constrain. Forecasts based only on local catalogues or short-term statistical extrapolation may consequently underestimate spatially distributed and post-injection responses. Statistical models can support rapid updating, but their performance depends on catalogue quality, sample size, magnitude thresholds and the temporal stability of injection. Physics-based models provide greater mechanistic insight, although they require geological and operational parameters that are rarely known with sufficient accuracy. Hybrid models can combine mechanistic constraints with adaptive statistical updating, while geomechanically informed machine learning may help identify complex relationships among geological conditions, injection operations, and seismic responses.

Because forecasting uncertainty cannot be eliminated, risk management should prioritize adaptive decisions over deterministic predictions. A geospatial susceptibility assessment can first identify areas where injection is relatively more likely to activate faults. These spatial estimates should then guide monitoring density, operational thresholds, and injection adjustments throughout the project life cycle. Within this framework, traffic-light systems serve as an operational response mechanism rather than a stand-alone safeguard.

## Figures and Tables

**Figure 1:**
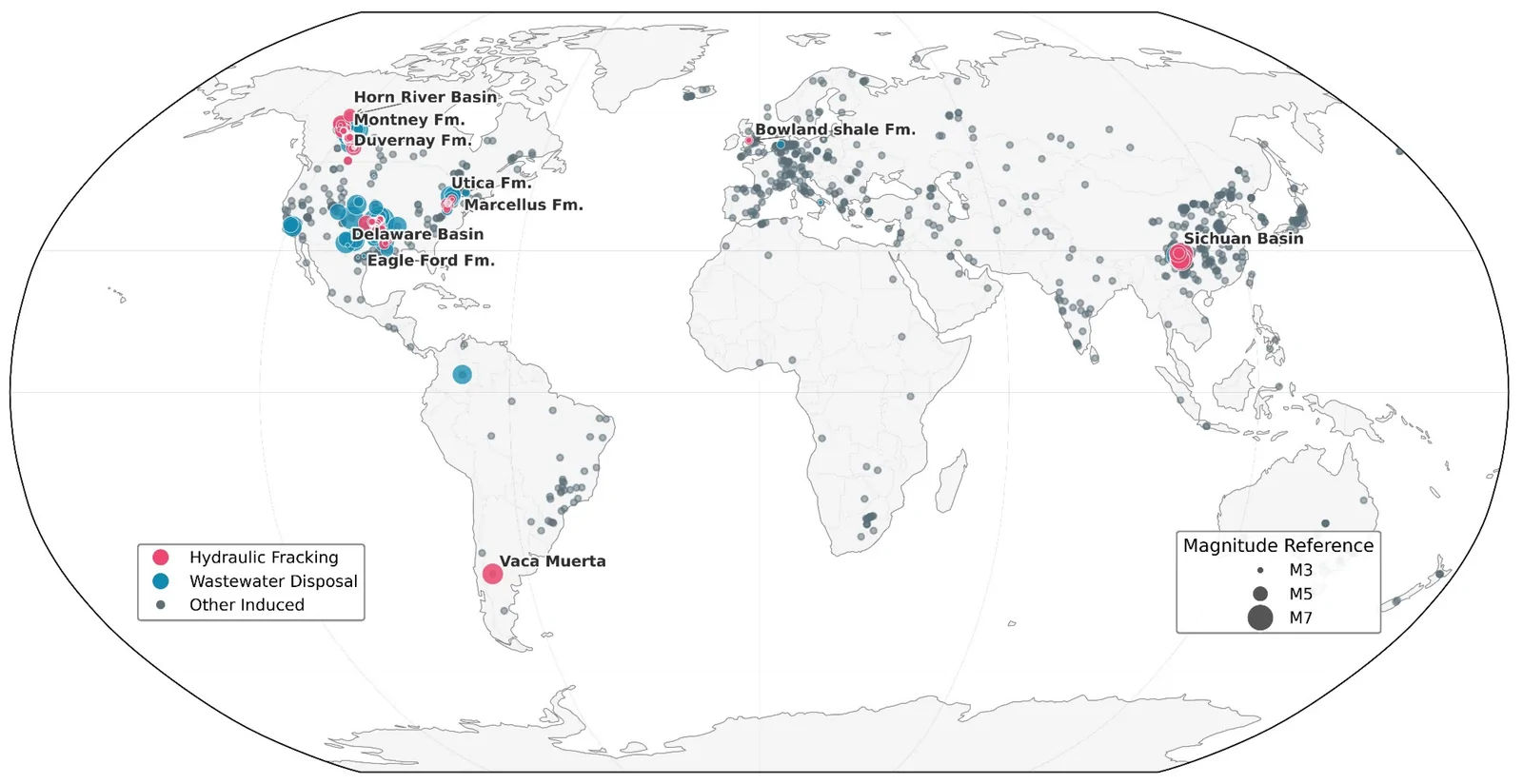
Global spatial distribution of shale-gas-related induced seismicity. Earthquake locations, activity classifications and reported maximum magnitudes are compiled from the Human-Induced Earthquake Database (HiQuake) [29]. Red and blue circles highlight cases classified as hydraulic fracturing and wastewater disposal, respectively, whereas grey circles show the background distribution of induced seismicity recorded in the database. Circle size indicates the reported maximum magnitude; M3, M5, and M7 are shown as reference sizes in the legend. Selected shale-gas basins and formations are labelled for geographical context. These activity-based classifications do not imply that all highlighted cases are directly associated with shale-gas development.

**Figure 2:**
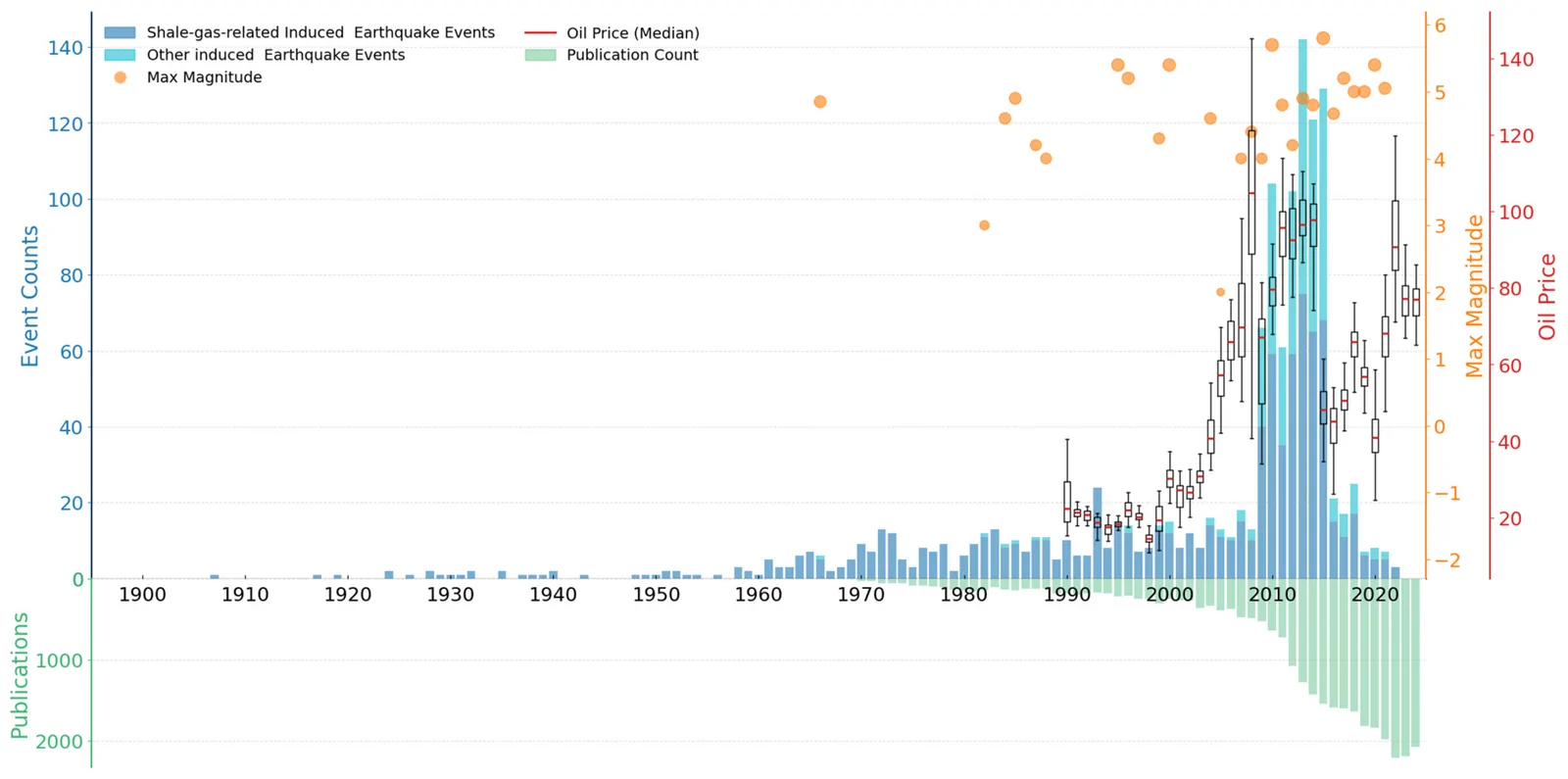
Temporal evolution of induced seismicity, oil-price variability and research activity related to shale-gas-induced seismicity. Blue bars show the annual number of shale-gas-related induced earthquake events from the Human-Induced Earthquake Database (HiQuake) [29], whereas cyan bars show other induced earthquake events in the same database. Orange dots indicate the reported maximum magnitude of shale-gas-related induced events for each year. Red boxplots show the annual distribution of crude oil prices based on historical West Texas Intermediate (WTI) futures data from 1990 to November 2024. Green bars indicate the annual Google Scholar search-result counts for publications from 1900 to 2024 retrieved using the combined keywords “shale gas” and “induced earthquake”. These counts are presented solely as a descriptive proxy for changes in research attention over time and were not used as the basis for selecting the literature included in this review.

**Figure 3:**
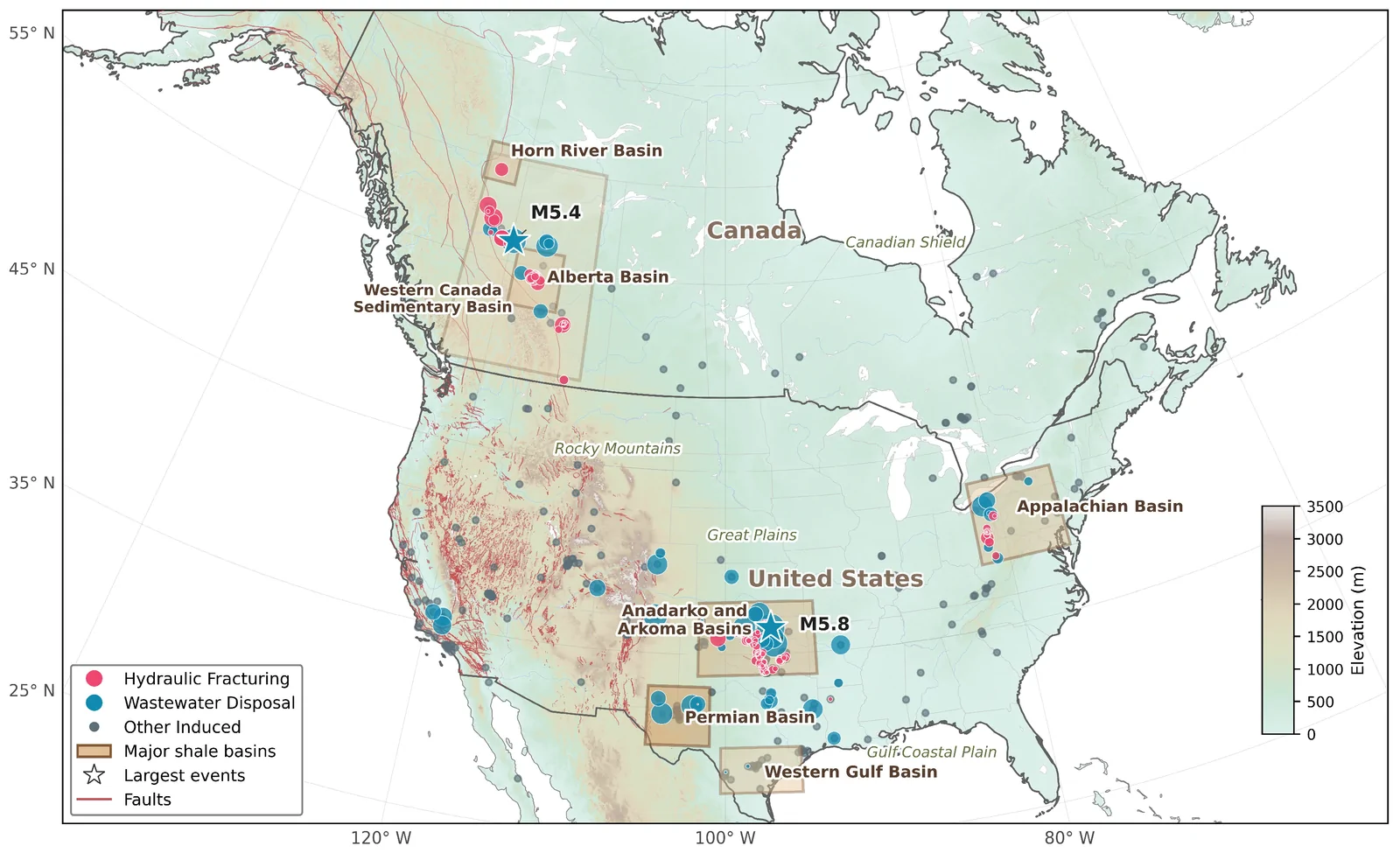
Spatial distribution of shale-gas-related induced seismicity in North America.

**Figure 4:**
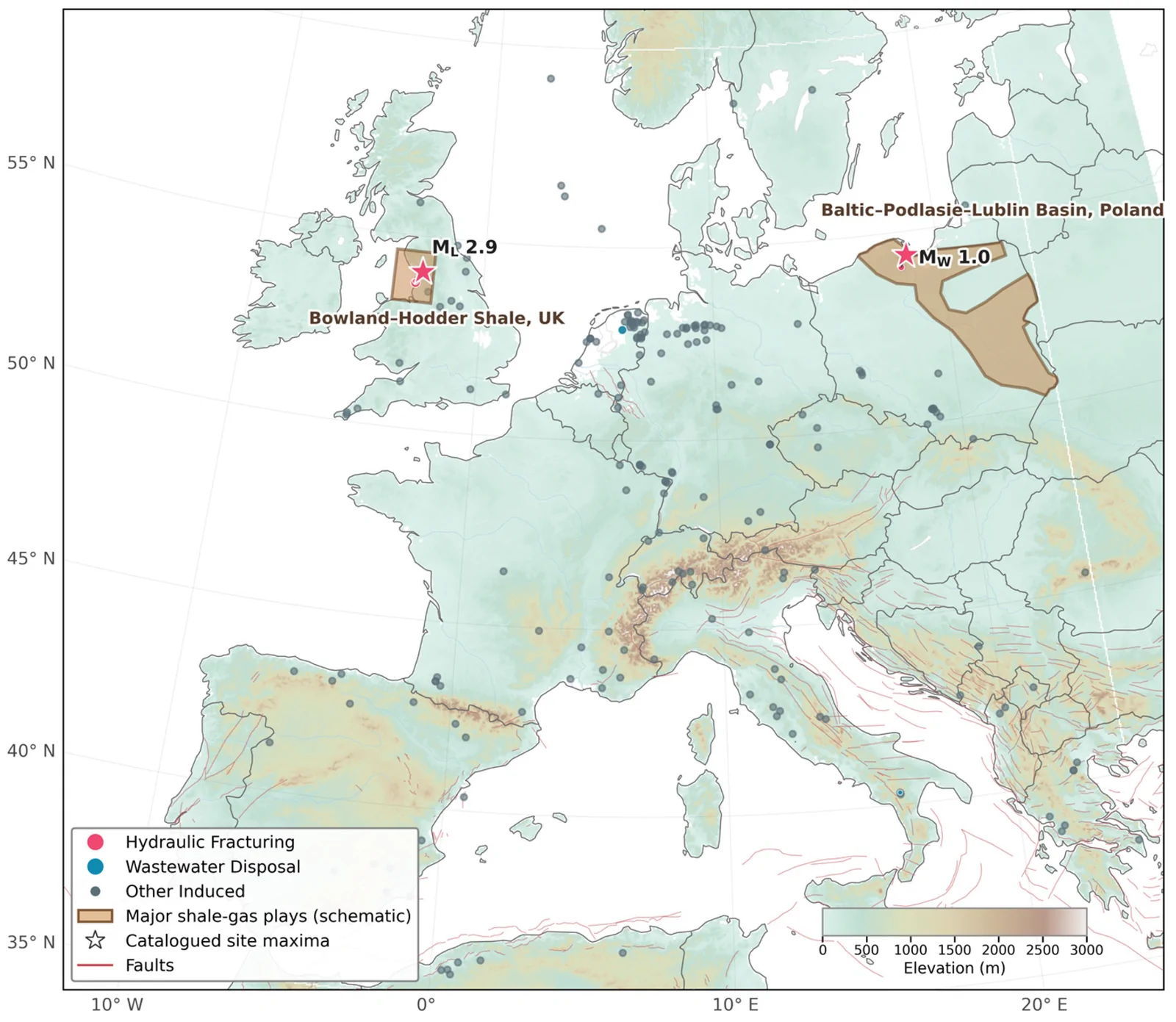
Spatial distribution of shale-gas-related induced seismicity in Europe.

**Figure 5:**
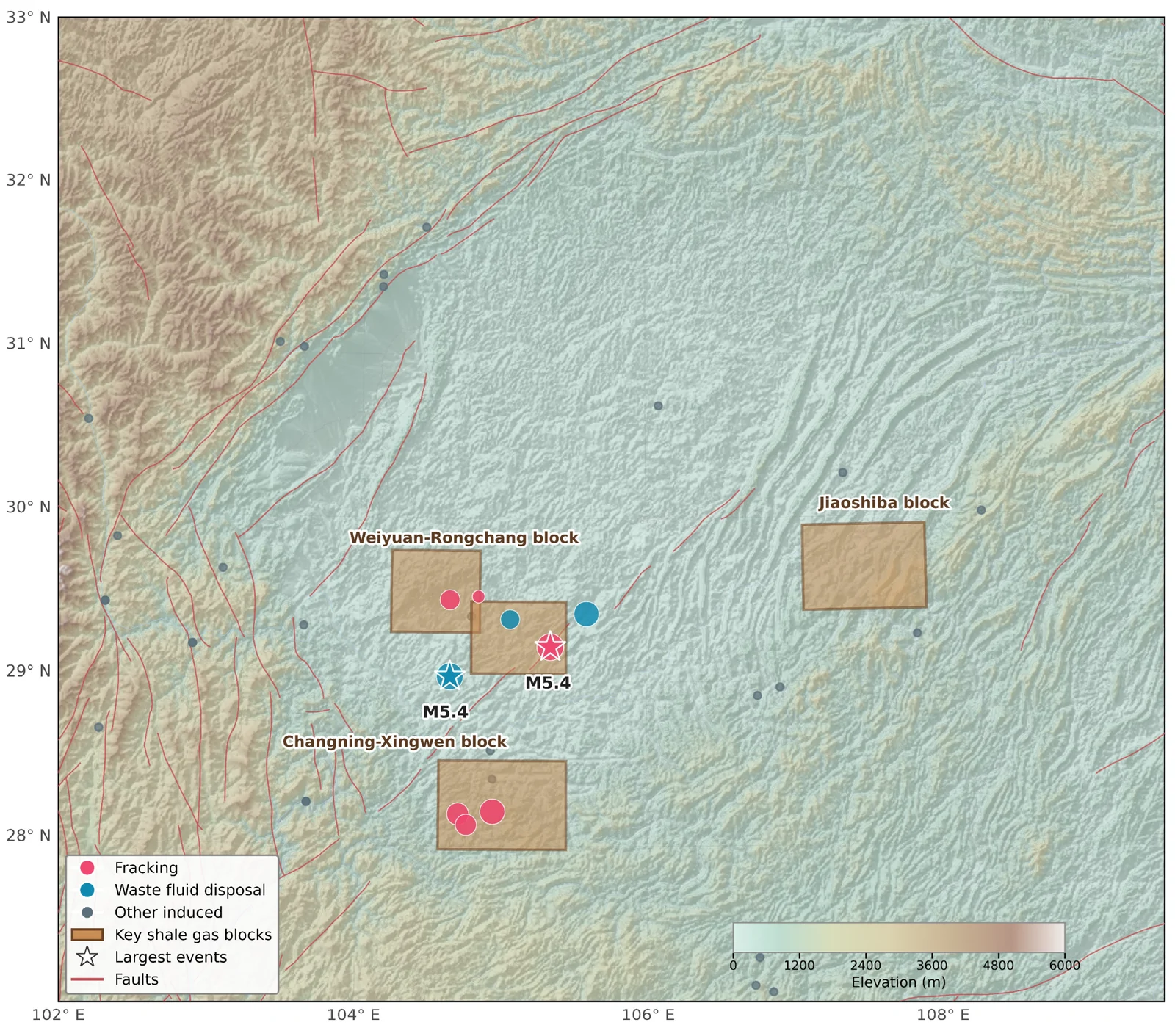
Spatial distribution of shale-gas-related induced seismicity in the Sichuan Basin, China.

**Figure 6:**
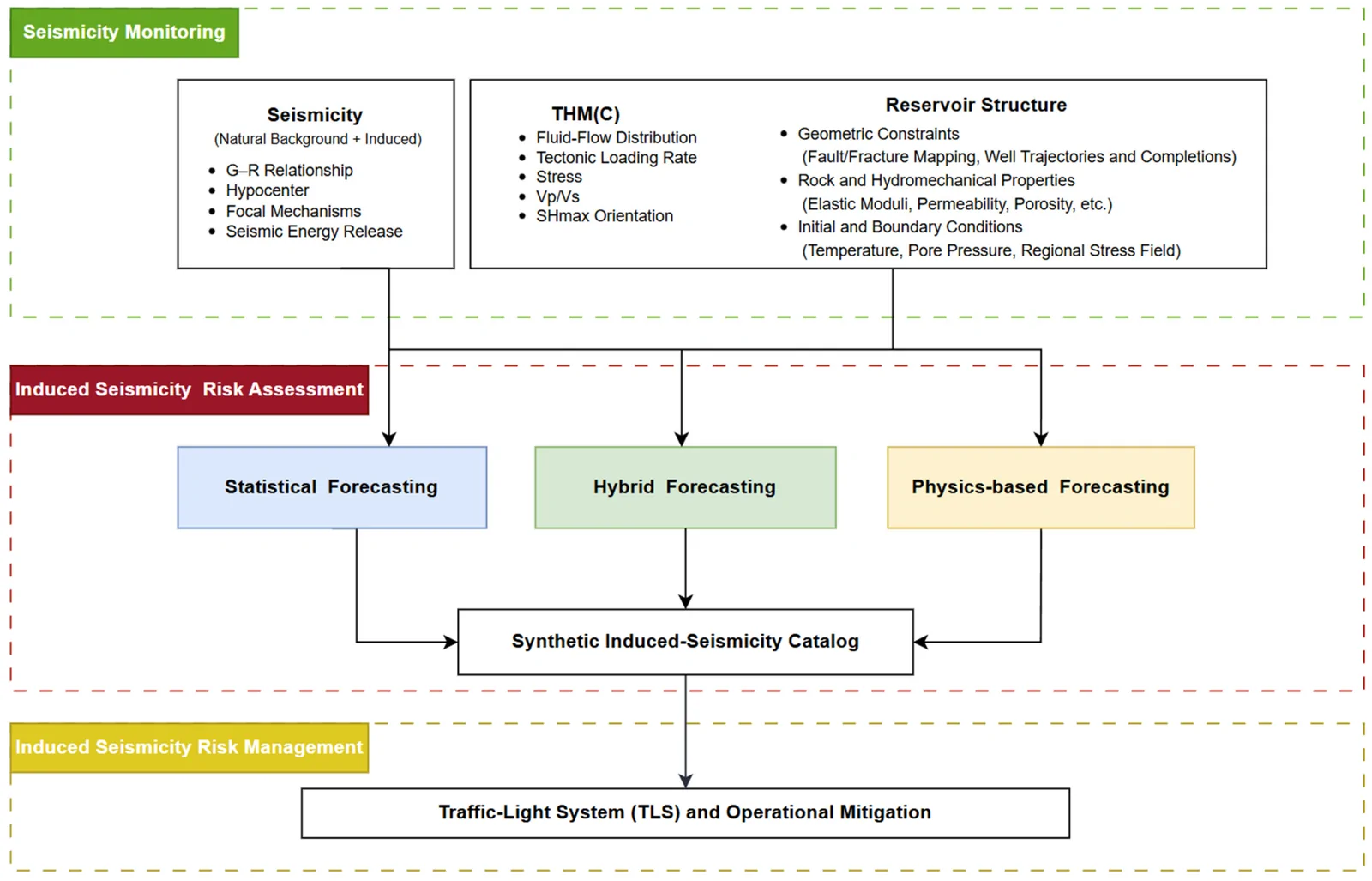
Integrated framework for monitoring, forecasting, and risk governance of shale-gas-induced seismicity (modified from [181]).

**Table 2:** Typical cases of shale-gas-related induced seismicity.

Operation Type	Country	Location	Maximum Induced Earthquake		References
			Magnitude	Date	
WD	USA	Youngstown, OH	$M_{w}3.9$	31 December 2011	[58]
		Dallas-Fort Worth, TX	$M_{b}$3.3	16 May 2009	[59]
		Raton Basin, CO and NM	$M_{w}$5.3	23 August 2011	[60,61,62]
		Prague, OK	$M_{w}$5.7	6 November 2011	[54,55]
		Timpson, TX	$M_{w}$5.0	17 May 2012	[63]
		Fairview, OK	$M_{w}$5.1	13 February 2016	[10,56]
		Pawnee, OK	$M_{w}$5.8	3 September 2016	[57]
		Cushing, OK	$M_{w}5.0$	7 November 2016	[57,64]
HF	USA	Garvin County, OK (Eola Field)	$M_{L}$2.9	18 January 2011	[65]
	Poland	Wysin Site	$M_{w}$1.0	13 August 2016	[28]
	Canada	Horn River Basin, BC	$M_{L}$3.8	19 May 2011	[50,66]
		Fort St. John, BC	$M_{w}$4.6	17 August 2015	[51,52]
		Fox Creek, AB	$M_{w}$4.1	12 January 2016	[67,68]
		Red Deer, AB	$M_{L}$4.2	4 March 2019	[69]
	UK	Preston New Road	$M_{L}$2.9	26 August 2019	[53,70]
Mixed	China	Rong County, Zigong, Sichuan	$M_{w}$4.6	25 February 2019	[18,71]
		Rongchang District, Chongqing	$M_{L}$5.1	10 September 2010	[72]
			$M_{L}$5.2	13 August 1997	[73,74]
		Xingwen County, Yibin, Sichuan	$M_{S}$5.7	16 December 2018	[18]
		Gong County, Yibin, Sichuan	$M_{S}$5.3	3 January 2019	[16,18]
		Changning County, Yibin, Sichuan	$M_{S}$6.0	17 June 2019	[75,76]
		Lu County, Luzhou, Sichuan	$M_{S}$6.0	15 September 2021	[18,77]

**Table 4:** Representative models, predictor variables, and outputs for shale-gas-related induced-seismicity forecasting.

Method Category	Representative Model	Core Equation or Relationship	Input Variables	Model Outputs	References
Statistical models	G–R relationship with finite-sample maximum-magnitude estimation	$M_{max}=M_{c}+(1/b)\;{log}_{10}\;N$	Magnitude of completeness ($M_{c}$); *b*-value; Number of events (*N*) with M≥Mc	$M_{max}$	[24]
	Injected-volume constraint on maximum seismic moment	$M_{0}^{max}\;\approx \;\mu \Delta V$	Shear modulus μ; Injected fluid volume ΔV	Conditional upper bound on *M_max_*	[23]
	ETAS and injection-rate-driven ETAS	$\lambda \left(t\right)=\mu_{inj}\left(t\right)+\lambda_{trig}\left(t\right)$	Earthquake catalogue; Injection rate	Seismicity rate	[178]
Physics-based models	Poroelastic and geomechanical fault-stability model	Injection-induced pore-pressure and poroelastic stress changes modify the effective stress and stability of pre-existing faults.	Injection history and well locations; Hydraulic and elastic properties; Initial stress state; Fault geometry and orientation	Pore-pressure and stress changes	[195]
Hybrid models	Linear poroelasticity coupled with rate-and-state friction	Injection-induced pore-pressure and poroelastic stress changes are linked to seismicity rates through a rate-and-state model.	Injection history and well locations; Hydraulic–elastic properties; Initial stress; Fault geometry and orientation; Friction parameters	Spatiotemporal seismicity rate	[25]
	Fluid-flow model coupled with a statistical seismicity model	Modelled pore-pressure increase rates are linked to seismicity rates through a spatially calibrated seismogenic index.	Injection history; Hydraulic properties; Pore-pressure increase rate; Spatial seismogenic index; Earthquake catalogue		[21]
	3-D poroelastic model coupled with a seismogenic-index model	Modelled Coulomb stressing rates are linked to seismicity rates through a spatially calibrated seismogenic index.	Injection history; Well locations; Hydraulic and elastic properties; Coulomb stressing rate; Spatial seismogenic index; earthquake catalog		[179]
Machine-learning models	Random Forest regression	Random Forest maps operational, geological, and simulated pressure–stress features to seismicity rates.	Injection rate and pressure; Geological attributes; Simulated poroelastic stresses	Seismicity rate; Predictor importance	[199]
	XGBoost with SHAP interpretation	XGBoost classification and regression models estimate whether hydraulic-fracturing pads induce earthquakes and how many events they produce; SHAP quantifies feature contributions.	Injected volume; Prior cumulative injection volume; Fracturing-stage counts; Well-pad location; Structural setting		[200]

**Table 5:** Regulatory measures for shale-gas-related induced seismicity.

Country	Region	Year	Regulatory Measures	References
Canada	British Columbia	2016	Ground motion monitoring is required, and a monitoring report shall be submitted within 30 days after the completion of operations. If an induced earthquake with $M_{L}>4.0$ occurs within 5 km of the operational well, hydraulic fracturing activities must be suspended.	[68]
		2018	A high-density accelerometer network was deployed within approximately 3 km of drilling platforms, lowering the reporting threshold for peak ground acceleration (PGA) from 2% *g* to 0.8% *g*.	[219]
	Alberta	2015	A traffic light system (TLS) was implemented, with the yellow- and red-light thresholds set at $M_{L}$2.0 and $M_{L}$4.0, respectively.	[220]
		2019	The traffic light thresholds were lowered, with the red- and yellow-light levels set at $M_{L}$3.0 and $M_{L}$1.0, respectively. In parallel, the Scientific Induced Seismicity Monitoring Network (SCISMN) was established to facilitate industry-wide waveform data sharing and to support regulatory decision-making.	[221]
United States	Arkansas	2012	Regional bans were imposed on wastewater disposal wells in areas of intensified seismic activity. In addition, permanent no-injection zones were designated near major fault systems, and selected existing disposal wells were permanently shut in.	[222]
	Ohio	2014	Seismic monitoring is required for proposed hydraulic fracturing wells located within approximately 4.8 km of known faults or areas of previous seismicity. Operational plans must be adjusted when earthquakes of $M_{L}$2.0~2.4 occur; operations shall be suspended and a risk assessment conducted when $M_{L\;}$≥ 2.5; and operations must be immediately halted when $M_{L}$ > 3.0.	[223]
	Oklahoma	2018	Within the designated area, operators monitor seismicity within 5 km of hydraulic fracturing. Responses escalate from the operator’s seismicity response plan ($2.0\leq M_{L}<2.5$), to notification/conditional six-hour pause ($2.5\leq M_{L}<3.0$), six-hour pause and consultation ($3.0\leq M_{L}<3.5$), and immediate suspension with in-person review ($M_{L}\geq 3.5$).	[216]
UK		2012	Upon detection of an earthquake with $M_{L}$ ≥ 0.5, hydraulic fracturing operations must be suspended, and resumption is permitted only after at least 18 h of continued monitoring and subsequent regulatory assessment.	[224]

## Data Availability

No new data were created or analyzed in this study. Data sharing is not applicable to this article.
